# Computer Vision Evidence Supporting Craniometric Alignment of Rat Brain Atlases to Streamline Expert-Guided, First-Order Migration of Hypothalamic Spatial Datasets Related to Behavioral Control

**DOI:** 10.3389/fnsys.2018.00007

**Published:** 2018-05-01

**Authors:** Arshad M. Khan, Jose G. Perez, Claire E. Wells, Olac Fuentes

**Affiliations:** ^1^UTEP Systems Neuroscience Laboratory, University of Texas at El Paso El Paso, TX, United States; ^2^Department of Biological Sciences, University of Texas at El Paso El Paso, TX, United States; ^3^BUILDing SCHOLARS Program, University of Texas at El Paso El Paso, TX, United States; ^4^Border Biomedical Research Center, University of Texas at El Paso El Paso, TX, United States; ^5^Department of Computer Science, University of Texas at El Paso El Paso, TX, United States; ^6^Graduate Program in Pathobiology, University of Texas at El Paso El Paso, TX, United States; ^7^Vision & Learning Lab, University of Texas at El Paso El Paso, TX, United States

**Keywords:** stereotaxic, stereotactic, atlas, data migration, registration, computer vision, subject matter expert, behavioral control

## Abstract

The rat has arguably the most widely studied brain among all animals, with numerous reference atlases for rat brain having been published since 1946. For example, many neuroscientists have used the atlases of Paxinos and Watson (*PW*, first published in 1982) or Swanson (*S*, first published in 1992) as guides to probe or map specific rat brain structures and their connections. Despite nearly three decades of contemporaneous publication, no independent attempt has been made to establish a basic framework that allows data mapped in *PW* to be placed in register with *S*, or vice versa. Such data migration would allow scientists to accurately contextualize neuroanatomical data mapped exclusively in only one atlas with data mapped in the other. Here, we provide a tool that allows levels from any of the seven published editions of atlases comprising three distinct *PW* reference spaces to be aligned to atlas levels from any of the four published editions representing *S* reference space. This alignment is based on registration of the anteroposterior stereotaxic coordinate (*z*) measured from the skull landmark, Bregma (β). Atlas level alignments performed along the *z* axis using one-dimensional Cleveland dot plots were in general agreement with alignments obtained independently using a custom-made computer vision application that utilized the scale-invariant feature transform (SIFT) and Random Sample Consensus (RANSAC) operation to compare regions of interest in photomicrographs of Nissl-stained tissue sections from the *PW* and *S* reference spaces. We show that *z*-aligned point source data (unpublished hypothalamic microinjection sites) can be migrated from *PW* to *S* space to a first-order approximation in the mediolateral and dorsoventral dimensions using anisotropic scaling of the vector-formatted atlas templates, together with expert-guided relocation of obvious outliers in the migrated datasets. The migrated data can be contextualized with other datasets mapped in *S* space, including neuronal cell bodies, axons, and chemoarchitecture; to generate data-constrained hypotheses difficult to formulate otherwise. The alignment strategies provided in this study constitute a basic starting point for first-order, user-guided data migration between *PW* and *S* reference spaces along three dimensions that is potentially extensible to other spatial reference systems for the rat brain.

## Introduction

Following the 1930s, when the design for the original Horsley-Clarke stereotaxic instrument (Horsley and Clarke, 1908) underwent modifications (Ranson and Ingram, 1931; Harrison, 1938) and was later diversified for performing intracranial surgery in the laboratory rat (Clark, 1939; Beattie, 1952; Stellar and Krause, 1954; Greer et al., 1955; Andreas and Legler, 1969; Krieg, 1975; also see Hillarp, 1947 for an alternate technology), several investigators published various stereotaxic coordinate systems to aid in the precise manipulation of small brain structures in this animal model, beginning with Krieg's atlas of 1946 (Krieg, 1946) (see Table 4 in Khan, 2013). Such manipulations have included ablation or stimulation of brain structures (Sheer, 1961; Myers, 1974; Thompson, 1978), tissue microdissection for biochemical analyses (Palkovits and Brownstein, 1988), chemical sampling of brain extracellular space via microdialysis or electrochemistry (Parada et al., 1998; also see Carter and Shieh, 2015), delineation of neural circuits using tracers (Heimer and Robards, 1981; Zaborszky and Heimer, 1989; Zaborszky et al., 2006), or molecular neurobiological techniques involving antisense, RNA interference, or viral-based vector delivery of various constructs to activate or silence activity in a cell-specific manner (Khan, 2013). More recently, such manipulations have also included optogenetic studies in rats (e.g., Gradinaru et al., 2009; Witten et al., 2011), including studies involving *in vivo* stimulation of hypothalamic cell bodies, their axonal projections, or their axonal inputs (Larson et al., 2015; Gigante et al., 2016), a structure that we also focus on in this study. Stereotaxic-based methods to manipulate brain structures to control behavior in the rat have contributed richly to our collective understanding of structure-function relations in the brain.

However, an inevitable outcome from these efforts—which collectively now span over seven decades of research using rat brain stereotaxic atlases—has been that anatomical data have been mapped within several different stereotaxic coordinate systems, hampering our abilities to interrelate formally the hard-earned and valuable results published in numerous studies. For example, the locations of injection sites published by a laboratory using a particular stereotaxic rat brain atlas may be difficult to place in register with corresponding locations, *within the same physical space*, of neuronal populations that might lie underneath such injections, but which have been mapped by another laboratory using a different stereotaxic atlas. This is because of several variables that will differ between such atlas reference spaces: plane of section, intervals between sections, originations of various “zero” points for Cartesian coordinates calibrated to landmarks on the skull surface, and strains and body weights of the animals used to create the atlases (Kruger et al., 1995; Khan, 2013). Indeed, the idea of “interoperability” between different software and hardware systems in computer science is now being extended to describe similar needs for anatomical reference frameworks of the brain (Zaslavsky et al., 2010; Hawrylycz et al., 2011), which have also been represented digitally in three-dimensional space (Toga et al., 1989, 1995; Timsari et al., 2001; Hjornevik et al., 2007).

The problem of poor interoperability is compounded further by the progression of time. Older editions of brain atlases fall out of fashion, go out of print, or are supplanted by more popular coordinate systems of other atlases, or by newer editions of the same atlas. Take, for instance, a laboratory that published critical data about a neural system two decades ago, using what were then state-of-the-art techniques to map their anatomical data to what was then a current edition of a specific rat brain stereotaxic atlas. Today, data from that study may no longer be so useful to laboratories that routinely use a different atlas reference space and entirely different coordinates based on a radically different plane of section. Thus, the high quality data from this 20 year-old study are now “trapped” within an old reference space, effectively sealed by coded locks that no longer have appropriately registered keys. The consequence of this is that if no other laboratory has taken up the same problem, those trapped data continue to represent all that is known about that particular structure-function relation in the brain, but our abilities to interpret that information continue to decrease with time. A related consequence is that current investigators may have to repeat the same experiment because they cannot contextualize such data with their own observations. These issues are similar to those envisioned over 75 years ago (Asimov, 1942), and also discussed in relation to the “Digital Dark Age(s),” in which older information may not be obsolete, but simply locked or uninterpretable, similar to software or hardware that no longer is accessible due to modernization of digital standards (Sanders, 1997; Rosenzweig, 2003; Lima, 2011; also see Lepore, 2015). The locked data may still be useful and relevant if there was a living key. Also, even if neuroanatomical data from a study are not yet “trapped,” migrating or registering them to additional anatomical reference spaces ensures their continued widespread use, lasting preservation, and broader contextualization with other (both older and newer) datasets [see, for example, the GitHub methods package release (https://github.com/RittmanResearch/maybrain) from Whitaker et al. (2016) to contextualize human brain MRI data with human brain gene expression data collected by the Allen Institute for Brain Sciences]. If supported by a durable and upgradable infrastructure, an extant anatomical reference space can serve as a stable repository and unified model for all spatial information concerning the brain of a particular species.

A viable solution to ensure that neuroanatomical datasets remain within living reference spaces is to make them as widely available as possible, a task that can be achieved in part by migrating the data to more than one living reference space. This strategy also affords scientists the ability to contextualize the migrated spatial dataset with unique resident datasets already mapped within the host atlas. Such migration could serve as a powerful means for investigators to formulate new hypotheses about diverse spatial datasets that they discover for the first time to be co-registered to the same region of the brain, a discovery process akin to the classic, albeit now mythologized, discoveries of the spatial relationships among cholera deaths, sewer access points and city water pumps by Edmund Cooper and John Snow using co-registered spatial datasets (Brody et al., 2000). As one of us has argued before (see section 4.6.2.2ff of Khan, 2013), co-registering datasets from experimental neuroscience studies, for example, to the same reference space allows investigators to formulate new ideas concerning the relationships between an experimental manipulation in the brain, the underlying neural substrates being manipulated, and the behavioral or physiological outcomes of such manipulation. Additionally, since the prevalence of numerous reference atlases stems partly from the need for atlas-makers to furnish their own interpretations about how brain structures are organized and parcellated, migration of a spatial dataset into a new host atlas allows investigators to place their results within the unique universe of discourse (Boole, 1854) of the host atlas creator, which could lead to new theoretical and/or empirical determinations of how the dataset contributes to our understanding of brain structure and function.

In this study, portions of which have been presented in preliminary form (Khan, 2013; Wells and Khan, 2013; Hernandez and Khan, 2016; Perez et al., 2017; Wells, 2017), we sought to fulfill three objectives: (1) to establish a basic alignment of 11 rat brain atlases based on their shared set of anteroposterior (AP) stereotaxic coordinates derived from the Bregma landmark on the skull surface; (2) to develop and implement a novel computer vision algorithm to independently provide evidence—from internal landmarks in the brain—about the usefulness of the basic AP stereotaxic alignment; (3) to migrate unpublished spatial datasets related to behavioral control experiments involving the hypothalamus, and in the process, determine whether expert-guided mapping would be required to migrate data from one atlas space to another in the mediolateral and dorsoventral dimensions.

## Materials and methods

### Creation of an anteroposterior alignment tool

#### Data entry and sorting

Coordinates based on the distance from the skull landmark, Bregma (β), in mm (hereafter designated as “β coordinate,” or *z*) listed for each of the 312 unique atlas levels from all editions of *PW* and *S* were entered manually into a spreadsheet (Microsoft Excel for Mac 2011, version 14.2.3; Microsoft Corp., Redman, WA). The numerical sequences of atlas levels for *PW* atlas editions fell within three separate groups: (1) a “1982/86/97” group (*PW1*) that is derived from the same tissue set and has identical atlas level assignments (Paxinos and Watson, 1982, 1986, 1997); (2) a “1998” group (*PW2*; Paxinos and Watson, 1998) that is derived from the same tissue set as *PW1*, but is assigned as a separate group because it includes from that tissue set two previously unpublished tissue sections and associated atlas drawings; thereby altering the numerical sequence of the atlas levels; and (3) a “2005/2007/2014” group (*PW3*) that is based on a tissue set with drawings and atlas levels completely distinct from *PW1* and *PW2* (Paxinos and Watson, 2005, 2007, 2014). These three *PW* groups were organized into separate columns, alongside a column containing *S* atlas levels (these are identical for all four editions and based on the same brain: Swanson, 1992, 1998, 2004, 2018), and a column of *z* values pooled from all 11 atlases (*PW*'82,'86,'97,'98,'05,'07,'14; *S*'92,'98,'04,'18). All atlas levels sharing the same coordinates were assigned to a common row within the spreadsheet.

#### Construction of dot plots

All atlas levels were to be calibrated to the same scale (*z*), and required separate plotting along this scale by atlas group. Thus, these levels needed to be plotted within one-dimensional rather than two- or three-dimensional (Cartesian) space (i.e., there are no *x*- or *y*-axis values for this dataset). For this purpose, two sets of resources were very helpful. First, the guidelines offered by Wilkinson (2005) and Carr and Pickle (2010) for the representation of one-dimensional data, along with the seminal papers of Cleveland (1984) and Cleveland and McGill (1984) on graphical perception theory, prompted the decision to represent the data as a set of Cleveland dot plots. Second, the online guidelines provided by O'Day for plotting climate change data (see O'Day, 2011) were very helpful to transform the raw data in Excel to such plots. Specifically, a dummy *y*-axis was created to numerically rank order distinct atlas level groups (see above) and then plot all atlas levels from these groups along a *z*-axis scale of β coordinate values. The arbitrary *y*-axis rankings were assigned specific atlas group labels to sort them, and the resulting graph of dot plots was re-drawn for publication using Adobe Illustrator Creative Suite 4 (Adobe Systems, Inc., San Jose, CA). Individual dots falling along the *z* scale in *PW* reference space (*z*_*PW*_) were color-coded on the basis of their proximity to dots in *S* space along the same scale (*z*_*S*_). Specifically, those levels where *z*_*PW*_ = *z*_*S*_ (and technically, where *z*_*S*_ = *z*_*PW*_) were coded as “*Fully in Register*”; those where *z*_*PW*_ – *z*_*S*_ ≤ 50 μm (or *z*_*S*_ – *z*_*PW*_ ≤ 50 μm) were coded as “*Narrowly in Register*”; and those where *z*_*PW*_ – *z*_*S*_ > 50 μm (or *z*_*S*_ – *z*_*PW*_ > 50 μm) were coded as “*Not in Register.”*

### Creation and implementation of a computer vision algorithm to compare atlas levels

To begin efforts toward automating the process of pairing *PW* and *S* atlas levels, we developed an algorithm to compare images of the Nissl-stained tissue accompanying the atlas levels and rank matches based on a similarity metric. For each image under analysis, the algorithm builds a descriptor by finding a set of local features that are invariant to changes in scale, illumination, and orientation, and partially invariant to geometric distortion. Given two images, their similarity is estimated by determining the number of local features that they have in common, subject to geometric constraints. Image descriptors are computed using the Scale Invariant Feature Transform (SIFT) (Lowe, 1999, 2004) while feature matching under geometric constraints is attained by applying the Random Sample Consensus (RANSAC) algorithm (Fischler and Bolles, 1981).

#### SIFT algorithm

After selecting a region of interest (ROI) from a given image, its features are computed and encoded using SIFT. The SIFT algorithm includes both a detector—which selects points of interest by finding high-contrast points that are maxima or minima of the difference of Gaussians in scale space for the ROI—and a descriptor, which encodes the selected points as a 128-dimensional feature vector describing the frequency distribution of the gradient orientations in a circular region surrounding the point of interest. Rotation invariance is attained by measuring all gradients with respect to the region's dominant orientation.

#### Matching

For every feature vector *u* in the descriptor of the ROI, we find the two most similar feature vectors *v* and *w* in the descriptor of the target image, according to their Euclidean distance |*u* – *v*|. If |*u* – *v*| is smaller than a predefined threshold, and the ratio |*u* – *v*|/|*u* – *w*| is less than 0.8, *u* and *v* are considered a match.

#### RANSAC

Once a set of matches between the ROI and an image is obtained, we find the largest subset of matches that are geometrically consistent. A set of matches *M* = {(*p*_1_, *q*_1_), (*p*_2_, *q*_2_),…,(*p*_*n*_, *q*_*n*_)}, where *p*_1_,…, *p*_*n*_ and *q*_1_,…,*q*_*n*_ are points of interest in the ROI and the target image, respectively, is geometrically consistent if there is an affine transformation or homography *H* such that *H*(*p*_*i*_) = *q*_*i*_, for 1 ≤ *i* ≤ n. To find the largest set of matches we use the RANSAC algorithm. RANSAC is a randomized iterative procedure that consists of the following steps: first, we randomly select from the set of matches the minimum number of matches required to compute a homography, which is four in this case. Then we compute the corresponding homography H and, for each match (*p*_*i*_, *q*_*i*_) in the set, we measure the reprojection error |*H*(*p*_*i*_) – *q*_*i*_|. If the error is less than 10 pixels, we consider the match correct and the point is labeled as an inlier, otherwise it is labeled as an outlier. This process is repeated for 2,000 iterations; at the end the homography with the largest number of inliers is retained and its corresponding number of inliers or geometrically consistent matches is considered the metric of similarity between the ROI and the candidate image.

**Figure 1:**
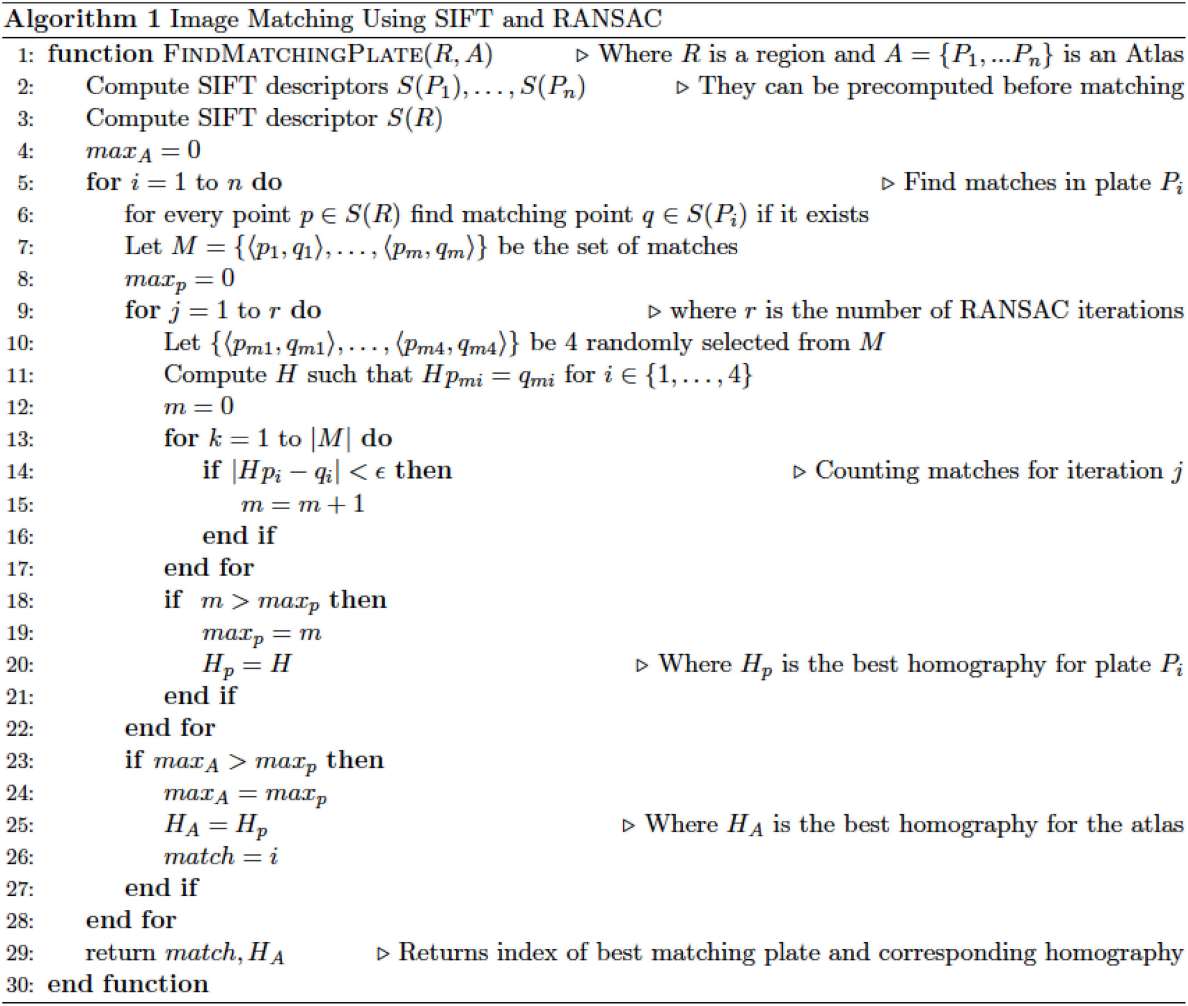
Pseudocode delineating the operations of the custom-made algorithm developed for this study, based on SIFT and RANSAC operations.

The whole process of feature extraction, matching, and homography search is repeated for every candidate image and the output is a list of images sorted by similarity to the ROI. The pseudocode in Figure 1 illustrates the complete process. The program was written in Python using OpenCV3 (Open Source Computer Vision Library; opencv.org), PyQt5, Scikit-Learn, SciPy, and NumPy; and is available for download at http://www.github.com/DeveloperJose/Vision-Rat-Brain.

#### Experiments to test algorithm

To test the computer vision algorithm, three experiments were conducted. *Experiment 1a*, which was essentially a proof of concept, was designed to task the algorithm to determine the Nissl image of origin within the Swanson atlas from where a test region of interest (ROI) was extracted. To implement this, a region of interest was extracted from Level 34 of *S* space, rotated 155 degrees, and distorted slightly using random point warping. It was then used to test the algorithm's ability to identify it as being part of Level 34's Nissl plate. Additionally, comparisons were performed to test the overall matching output before and after the RANSAC module of the algorithm was applied. In *Experiment 1b*, a comparison test was performed to determine the algorithm's ability to recognize the source of an undistorted test ROI from L34 as originating from the Nissl photomicrograph of L34 as opposed to a photomicrograph from a different level of the *S* atlas. In *Experiment 2*, the ability of the algorithm to recognize the appropriately matching plate from *S* space, which corresponds to a test ROI extracted from a Nissl image from *PW* space, was evaluated. For *Experiments 1b and 2*, the number of SIFT matches and RANSAC inliers was computed by the algorithm and the results tabulated in rank order with the highest ranking match being the solution associated with the highest number of SIFT matches and RANSAC inliers.

### Data transformation

#### Transformation of unpublished experimental data

Since the injection site locations for experiments from a published behavioral study for the hypothalamus (Khan et al., 2004) had not been included in that publication, we decided to re-visit this dataset and use it to test our data transformation and migration methods and, in the process, place some of these sites in the published record within an atlas reference space. The goal of this exercise was to illustrate how an unpublished dataset could be migrated into an atlas reference space years after it had been generated. Below, each step is described in detail to aid readers in their own attempts to update and unlock older datasets. For the process described here, the data to be migrated were originally mapped into a reference space for which digital formats did not exist, requiring first a series of transformations to migrate them to vector-formatted space.

##### Mapping injection sites in PW space (histological to graphical transformation)

In the behavioral experiments reported by Khan et al. (2004), adult male Sprague-Dawley rats (350–500 g BW) received stereotaxic implantations of chronic indwelling stainless steel guide cannulas targeting the LHA. The stereotaxic coordinates were: +6.1–6.4 mm anterior to the interaural line, +1.8 mm lateral to the midsagittal sinus, and –8.2 mm ventral to the skull surface; with the incisor bar set at −3.3 mm (Paxinos and Watson, 1986). The injection sites were originally preserved in tissue and mapped as follows. Tissue was prepared by transcardially perfusing each subject with 10% formalin. After removal from the skull, brains were stored in 10% formalin at room temperature until sectioned, at which time they were blocked and frozen in powdered dry ice. The portion of the hypothalamus containing the injection site was cut into 100 μm-thick sections on the freezing stage of a Reichert sliding microtome. Sections were collected through the full extent of the injector needle track and injection site. Sections were mounted onto glass slides, air dried, stained with thionin, dehydrated in an ascending series of ethanol concentrations, cleared in xylene, and coverslipped using Permount or DPX. Selected slides containing thionin-stained sections with regions of interest were each mounted onto the stage of a Bausch & Lomb Microprojector™ projection microscope (Bausch & Lomb, Inc., Rochester, NY). The projected image of the tissue section containing the injection site was traced onto a size-adjusted, cropped, paper photocopy of the relevant figure from the second edition of the Paxinos and Watson rat brain atlas (Paxinos and Watson, 1986; *PW86*). The selection of each figure was determined by visual comparison of the Nissl-stained regions in the tissue with those found in the atlas photomicrographic plate accompanying each figure.

##### Graphical to digital transformation

Twelve to fifteen years after they were originally drawn, a total of 183 of the manually produced tracings on their selected *PW86* maps were bulk-scanned as digital images and imported into a vector graphics editor [Adobe Illustrator (AI) CS5, Adobe Systems Inc., San Jose, CA]. Each digital image was a composite scan that included (a) the traced outline of the injection site, and (b) the underlying map from *PW86*. Composite scans of injection site cases were imported into a common .ai file if the map onto which they were traced was at the identical anteroposterior (AP) stereotaxic coordinate (*z*). For example, if the composite scan was obtained for an injection site traced onto a photocopy of Figure 25 of *PW86* (*z* = –1.80 mm), it was imported into an .ai file along with other such composite scans for *z* = –1.80 mm. In the file, each scan resided on a separate transparent layer, and each .ai file therefore contained a set of injection sites that had been localized in the AP axis to the same atlas level.

##### Raster to vector transformation

The next step in the transformation of our data was to separate the injection site tracings from the underlying *PW* atlas plates onto which they were traced, which required that the separate components within each composite digital scan (tracings, underlying maps) were rendered into vector objects. The first and second editions of the Paxinos and Watson atlas (*PW82, PW86*) do not include electronic versions of the atlas plates, but the third edition (*PW98*) does. Since *PW86* and *PW98* reference spaces differ only in slightly revised drawings and the inclusion of two additional plates in the latter space, but are both derived from the same set of animal subjects and hence the same brains, we selected to trace the imported composite scans into a vector format using the *PW98* digital atlas maps as a template. For this purpose, the digital file from the *PW98* atlas that corresponded to the *PW86* map within the composite scan was imported into the .ai file as a separate layer, and aligned with the raster image of the scan. This was done such that registration of structures at and immediately surrounding the lateral hypothalamus (“LH” in *PW98*) was maximized, at the expense of the alignment of more distal structures. The *Pencil Tool* was used to trace over the injection site drawing on an additional transparent layer, thereby producing a vector drawing over the raster outline. A different transparent layer was created for each injection site, so long as all were mapped to the same atlas level within the given .ai file. The result of this effort was a single .ai file containing a *PW98* atlas layer and separate layers of injection site drawings in vector format, each drawn over the *PW98* layer and which could be visualized together or separately with the other injection sites, depending on whether the visibility of each layer was toggled on or off.

The format of the dataset produced from the procedures just described can be summarized as follows. Vector-formatted drawings of each injection site for a common AP level (*z*) of *PW98* were now present as individual transparent layers within a single .ai file. Importantly, the injection site outline was now digitally separated from its original *PW86* photocopied map and on a separate *PW98* layer in the .ai file. The benefit of this arrangement was that each injection site outline existed as a separate 2-D object that could be overlaid onto a separate data layer representing *PW98* space. The dataset was therefore now amenable for data migration (described next), since this would entail importing the comparable Swanson (*S*) atlas map into this stack of layers, permitting alignment of injection sites to *PW* space and also to *S* space.

#### Migration of experimental data

##### Data alignment

**Figure 2:**
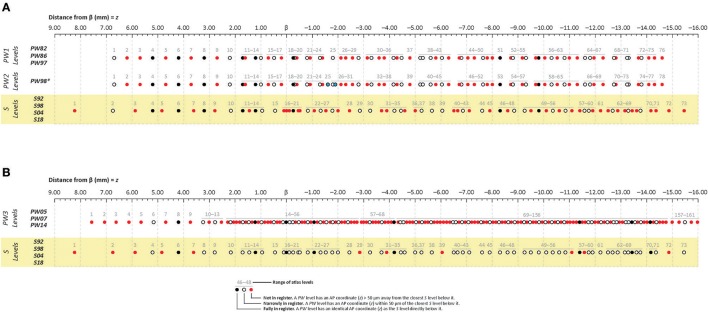
Cleveland dot plot charts illustrating craniometric alignments of sequential levels for Paxinos & Watson (*PW*) reference atlases with the Swanson (*S*) reference atlas. The charts are calibrated to a millimeter scale (found at the top of **A,B**) denoting anteroposterior (AP) distance from the cranial suture-based landmark, Bregma (β). The legend at the bottom of the figure defines each symbol, with filled black dots, filled red dots, and open circles in *PW* spaces denoting levels that are fully in register, not in register, or narrowly in register; respectively, with the corresponding dots directly below them in *S* space. **(A)** Cleveland plots aligning the atlas levels of two *PW* reference spaces (“*PW1 Levels”* and “*PW2 Levels”*) to *S* reference space (“*S Levels”*). “*PW1 Levels”* denote atlas levels from the first three editions of *The Rat Brain in Stereotaxic Coordinates* by Paxinos and Watson (1982, 1986, 1997) and are designated “*PW82*,” “*PW86*,” and “*PW97*,” respectively. “*PW2 Levels”* denote atlas levels from the fourth edition of *The Rat Brain in Stereotaxic Coordinates* by Paxinos and Watson (1998), which is designated “*PW98*.” Editions 2–4 contain refinements of the atlas drawings in the first edition, but the actual tissue sections on which the drawings are based are the same as those used in the original edition. *The only exception to this rule is that “*PW98*.” differs from “*PW82*,” “*PW86*,” and “*PW97*” in the addition of two levels from the original tissue set that had not been published in the earlier editions. These are highlighted as filled blue dots. Because these additions alter the numbering scheme for the “*PW98*” levels from those of previous *PW* editions, they have been displayed separately from those editions. **(B)** Cleveland plots aligning the atlas levels of a third *PW* reference space (“*PW3 Levels”*) to *S* reference space (“*S Levels”*). “*PW3 Levels”* denote atlas levels from the fifth, sixth and seventh editions of *The Rat Brain in Stereotaxic Coordinates* by Paxinos and Watson (2005, 2007, 2014) and are designated “*PW05*,” “*PW07*,” and “*PW14*,” respectively. They are in a separate reference space because the tissue used was from a different animal than that used for the earlier editions, which were actually based on tissue sections from several animals. **(A,B)** “*S Levels*” comprise atlas levels from all four editions of *Brain Maps: The Structure of the Rat Brain* by Larry W. Swanson, published in 1992, 1998, 2004, and 2018 (designated “*S92*,” “*S98*,” “*S04*,” and “*S18*”; respectively). They are all within one reference space because the same tissue set has been used for each edition, with the editions differing primarily in the refinement of the drawings and cytoarchitecturally derived mapped sub-regions from this single tissue set.

The alignment tool shown in Figure 2 was used to identify the levels of the Swanson (2004) atlas (*S* space) corresponding to the *PW98* levels, and the electronic version of the appropriate *S* atlas level was imported as a separate layer into each file and centered in the horizontal axis. The agreement of the *S* and *PW* maps was inspected visually and additional *S* maps anterior or posterior to the first map were also imported and centered as appropriate to correct for dorsoventral plane of section differences between the atlases. Mediolateral plane of section differences were less than one level (although this determination is complicated by the fact that only one hemisphere of the brain is mapped in the *S* atlas). The imported *S* atlas maps were cropped and combined to create a single *S* atlas “composite map” for each file.

##### Anisotropic scaling of atlas plates

For the levels of *PW98* used for this exercise (Figures 26, 31, and 33 of Paxinos and Watson, 1998), the *S* map was scaled anisotropically such that the digitally represented stereotaxic coordinate grid in the AI environment exactly matched that of the *PW* map. (This can also be achieved, in our experience, by using the scaling factors provided by (Swanson, 1992): 139% in the horizontal axis and 161% in the vertical axis; and then normalizing the proportions using a scaling factor of 139.5% in both axes on *PW*; the difference between these two methods of scaling the *S* map was 0.4% in the horizontal axis and 0.1% in the vertical axis).

##### Final transformation and migration of experimental data

After various warping methods (Wells, 2017) yielded limited success, it was determined that 2-D drawings needed to be represented as point-source data to enable accurate migration of the locations of the injection sites. Each injection site was approximated as a point-source datum by placing a circle upon its ventral margin in Adobe Illustrator, as near to the site's midline as possible, which corresponded to the ventral tip of each injection site. Toggling the visibility of the relevant layers in the .ai file permitted the point-source sites to be displayed on the *S* map in the file. Two steps were then conducted sequentially to migrate the data. First, the locations of the point source data in *PW* space were migrated to their directly corresponding stereotaxic locations in *S* space. Because this approach yielded several outliers that did not migrate to lawful locations in *S* space on the basis of stereotaxic coordinates alone, a second step was employed. Specifically, each site was shifted—in a subject expert-guided fashion—to the appropriate location in *S* space so that the original relationship between the site and nearby fiducials on the *PW* plate was recapitulated as closely as possible on the *S* map. A total of 24 injection sites were migrated in this study (**Table 7**), 20 of which were from experiments described in detail in Khan et al. (2004).

##### Quantitative analysis of data migration procedures

*Converting the destination atlas reference space to a Cartesian workspace*. In order to compute the errors in migrating point-source data prior to the step where expert-guided corrections were implemented, the mapped sectors covering the locations in *S* space where the data were migrated were treated as quadrants of a 2 × 2 mm Cartesian plane, with the ordinate defined as the dorsoventral axis, the abscissa as the mediolateral axis, and their intersection as origin *O* at (0,0) mm. A separate Cartesian plane was constructed in Adobe Illustrator (AI) for each point-source dataset in *PW*98 that was migrated to a unique *S* level. Thus, three separate Cartesian planes were constructed in AI, for *PW*98_26_
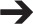
*S*_26_, *PW*98_31_
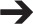
*S*_29_, and *PW*98_33_
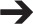
*S*_30_ injection site migrations, respectively.

*Error calculations*. Within the AI environment, the Cartesian plane, the migrated data points prior to expert-guided correction, and the data points relocated after expert-guided correction; were all placed on separate layers so they could be toggled visible or invisible as needed for the analysis. Millimeter units were assigned in the *Preferences*, rulers were toggled to visible, and the ruler origin was dragged to align precisely with the origin of the Cartesian plane being analyzed. With this arrangement, the *x* and *y* positions of each point-source datum could be queried by using the *Selection Tool* to select an individual data point and then consulting the *Info* window for specific positional information for the data point, expressed in mm from *O*. The *x* and *y* positions of the original (uncorrected) migrated data points were tabulated in an Excel spreadsheet in relation to the positions of the expert-guided (relocated) data points, and the differences in position calculated by subtracting the value of each uncorrected coordinate from the value of its corresponding relocated point. The mean and SEM for the errors in position along the *x* (ML) and *y* (DV) axes were calculated across migrated levels.

*Analysis of expert-guided corrections to the migrated datasets*. In order to compute the magnitudes and directions of the corrections performed by the subject expert on the migrated datasets, an additional layer within our Cartesian workspace in AI was created. In this layer, vectors were drawn from each pair of original and relocated data points in order to prepare diagrams showing the nature of the corrections performed by the subject matter expert. To compute the magnitude of each vector (AB), the numerical difference in the position of each original (A_*x*_, A_*y*_) and each relocated (B_*x*_, B_*y*_) data point was used to calculate the positional differences of vector AB along each axis (AB_*x*_ and AB_*y*_). The vector AB was computed by taking the square root of AB_*x*_^2^ + AB_*y*_^2^, and the direction of AB computed by calculating the arctangent (in radians) of AB_*x*_ and AB_*y*_. To this end, the two-argument variant of the arctangent was used (ATAN2 function in Excel) rather than the one-argument variant (ATAN), in order to have the calculation take into account the signs of both positions when assigning the direction to a specific quadrant. The resulting value was then converted to degrees to obtain φ, the final direction for AB. The mean magnitude and direction for all the vectors was then calculated to obtain the general behavior of the subject expert in correcting the migration of the datasets as a whole.

## Results

### Anteroposterior alignments based on craniometric measures using cleveland dot plots

To facilitate anteroposterior (AP) alignment between *PW* and *S* reference spaces, atlas levels corresponding to values along the *z*-axis (expressed as distance in mm from Bregma) were tabulated for all editions of each reference space and compared (Table 1). From these values, Cleveland dot plots were generated for the tabulated data as described in the Methods. Figure 2 presents Cleveland dot plots for all atlas levels of *PW* and *S* editions alongside one another. Comparisons between early *PW* atlas editions (“*PW1 Levels”* and “*PW2 Levels”*) and *S* atlas editions (“*S Levels”*) are made in Figure 2A, whereas later *PW* editions (“*PW3 Levels”*), based on a new set of tissue, are compared against *S* atlas editions (again, “*S Levels”*) in Figure 2B. The colored symbols in Figure 2 specify which levels between these reference spaces are *fully in register* (*z*_*PW*_ = *z*_*S*_; black dots), *narrowly in register* (|*z*_*PW*_ – *z*_*S*_| ≤ 50 μm (or | *z*_*S*_ – *z*_*PW*_| ≤ 50 μm); open circles), or *not in register* (|*z*_*PW*_ – *z*_*S*_| > 50 μm (or | *z*_*S*_ – *z*_*PW*_| > 50 μm); red dots) with one another along *z*.

**Table 1:** Alignments of *PW* atlas levels (Groups 1–3) with *S* atlas levels (Group 4) by distance from β.

	***PW***			***S***		***PW***			***S***		***PW***			***S***		***PW***			***S***		***PW***			***S***
***z*** **(mm)**	**1**	**2**	**3**	**4**	***z*** **(mm)**	**1**	**2**	**3**	**4**	***z*** **(mm)**	**1**	**2**	**3**	**4**	***z*** **(mm)**	**1**	**2**	**3**	**4**	***z*** **(mm)**	**1**	**2**	**3**	**4**
8.24				1	−0.11				18	−3.80	33	35			−7.68			97		−11.64			130	
7.56			1		−0.12			34		−3.84			65		−7.80	49	51	98		−11.75				59
7.08			2		−0.24			35		−3.90				32	−7.90				45	−11.76			131	
6.60			3		−0.26	18	18		19	−3.96			66		−7.92			99		−11.80	65	67		
6.74				2	−0.30	19	19			−4.08			67		−8.00	50	52			−11.88			132	
6.70	1	1			−0.36			36		−4.16	34	36			−8.04			100		−11.90				60
6.20	2	2			−0.40	20	20			−4.20			68	33	−8.16			101		−11.96	66	68		
6.12			4		−0.46				20	−4.30	35	37			−8.28			102		−12.00			133	
5.88				3	−0.48			37		−4.36			69		−8.30	51	53		46	−12.12			134	
5.70	3	3			−0.51				21	−4.44			70		−8.40			103		−12.20				61
5.64			5		−0.60			38		−4.45				34	−8.52			104		−12.24			135	
5.20	4	4		4	−0.72			39		−4.48	36	38			−8.60				47	−12.30	67	69		
5.16			6		−0.80	21	21			−4.56			71		−8.64			105		−12.36			136	
4.85				5	−0.83				22	−4.60				35	−8.72	52	54			−12.48			137	
4.70	5	5			−0.84			40		−4.68			72		−8.76			106		−12.50				62
4.68			7		−0.92	22	22			−4.80	37	39	73		−8.80	53	55			−12.60			138	
4.20	6	6	8	6	−0.96			41		−4.92			74		−8.85				48	−12.68				63
3.72			9		−1.08			42	23	−5.00				36	−8.88			107		−12.72	68	70	139	
3.70	7	7			−1.20			43		−5.04			75		−9.00			108		−12.80	69	71		
3.60				7	−1.30	23	23			−5.16			76		−9.12			109		−12.84			140	
3.24			10		−1.32			44		−5.20	38	40			−9.16	54	56			−12.88				64
3.20	8	8		8	−1.33				24	−5.25				37	−9.24			110		−12.96			141	
3.00			11		−1.40	24	24			−5.28			77		−9.25				49	−13.08			142	
2.80				9	−1.44			45		−5.30	39	41			−9.30	55	57			−13.15				65
2.76			12		−1.53				25	−5.40			78		−9.36			111		−13.20			143	
2.70	9	9			−1.56			46		−5.52			79		−9.48			112		−13.24	70	72		
2.52			13		−1.60		25			−5.60	40	42			−9.50				50	−13.28				66
2.28			14		−1.72			47		−5.64			80		−9.60			113		−13.30	71	73		
2.20	10	10			−1.78				26	−5.65				38	−9.68	56	58			−13.32			144	
2.16			15		−1.80	25	26	48		−5.76			81		−9.72			114		−13.44			145	67
2.15				10	−1.88		27			−5.80	41	43			−9.80	57	59		51	−13.56			146	
2.04			16		−1.92			49		−5.88			82		−9.84			115		−13.60				68
1.92			17		−2.00				27	−6.00			83		−9.96			116		−13.68	72	74	147	
1.80			18		−2.04			50		−6.04	42	44			−10.04	58	60			−13.76				69
1.70	11	11		11	−2.12	26	28			−6.06				39	−10.08			117		−13.80	73	75	148	
1.68			19		−2.16			51		−6.12			84		−10.10				52	−13.92			149	
1.60	12	12			−2.28			52		−6.24			85		−10.20			118		−14.04			150	
1.56			20		−2.30	27	29			−6.30	43	45			−10.30	59	61			−14.08	74	76		
1.45				12	−2.40			53		−6.36			86		−10.32			119		−14.16			151	70
1.44			21		−2.45				28	−6.48			87		−10.35				53	−14.28			152	
1.32			22		−2.52			54		−6.50				40	−10.44			120		−14.30	75	77		
1.20	13	13	23	13	−2.56	28	30			−6.60			88		−10.52	60	62			−14.36				71
1.08			24		−2.64			55		−6.65				41	−10.56			121		−14.40			153	
1.00	14	14			−2.76			56		−6.72	44	46	89		−10.60				54	−14.52			154	
0.96			25		−2.80	29	31			−6.80	45	47			−10.68			122		−14.60	76	78		
0.95				14	−2.85				29	−6.84			90		−10.80	61	63	123		−14.64			155	
0.84			26		−2.92			57		−6.85				42	−10.85				55	−14.76			156	
0.72			27		−3.00			58		−6.96			91		−10.92			124		−14.86				72
0.70	15	15			−3.12			59		−7.04	46	48			−11.00	62	64			−15.00			157	
0.60			28		−3.14	30	32			−7.08			92		−11.04			117		−15.24			158	
0.48	16	16	29		−3.24			60		−7.10				43	−11.10				52	−15.46				73
0.45				15	−3.25				30	−7.20			93		−11.16			118		−15.48			159	
0.36			30		−3.30	31	33			−7.30	47	49			−11.28					−15.72			160	
0.24			31		−3.36			61		−7.32			94		−11.30	63	65	119		−15.96			161	
0.20	17	17			−3.48			62		−7.44			95		−11.40				53					
0.12			32		−3.60	32	34	63		−7.56			96		−11.52			129						
0.10				16	−3.70				31	−7.60				44	−11.58				58					
0.00			33	17	−3.72			64		−7.64	48	50			−11.60	64	66							

*PW, Paxinos and Watson atlas levels; S, Swanson atlas levels. The numbers 1–3 denote different PW atlas groups, as described in the Methods: Group 1 = PW atlases published in 1982, 1986, and 1997. Group 2 = PW atlas published in 1998. Group 3 = PW atlases published in 2005, 2007, and 2014. All Swanson atlas editions (1992, 1998, 2004, 2018) are within Group 4. These tabulated data are represented using dot plots in Figure 2. Note that the Bregma values were measured directly by Paxinos & Watson for their (PW) atlases using stereotaxic procedures; these values have been used by Swanson to derive Bregma value estimates for his (S) atlases*.

### Differences in atlas level registration based on craniometric measures

When calibrated along *z*, only eight atlas levels were *fully in register* between *PW1 Levels*, and *S* levels and between *PW2 Levels* and *S Levels* (Figure 2A: black dots; Table 2). Similarly, only eight atlas levels were *fully in register* between *PW3 Levels* and *S Levels* (Figure 2B: black dots; Table 3). In contrast, there were several more *PW* atlas levels that were *narrowly in register* with corresponding *S* levels; in some cases, the distance separating the levels was as little as 10 μm (Figures 2A,B: white circles; Tables 4, 5). Since the atlases of the *PW3* group are based on a brain that was sampled at higher spatial resolution than those of the *PW1* and *PW2* groups (at primarily 120 μm intervals instead of 500 μm intervals; see the dense pattern of dots that reflects this fine-grained sampling for *PW3 Levels* in Figure 2B), registration of the atlas levels from this group with *S Levels* resulted in much greater numbers of *narrowly in register* pairs of atlas levels between the reference spaces. This is evident at a glance when examining the dot plots for *S* space in Figure 2: many more *not in register* levels (*red dots*) exist in *S* space in Panel A than in Panel B, where the red dots have been converted to white dots to denote their updated status as *narrowly in register* with the newer *PW3* reference space.

**Table 2:** *PW1* and *PW2* levels fully in register with *S* levels along the AP axis (*z* mm from Bregma).

***z***	***PW*** **Atlas Level**		***S* Atlas Level**
	***PW1***	***PW2***	***S92, S98, S04, S18***
+5.20	4	4	4
+4.20	6	6	6
+3.20	8	8	8
+1.70	11	11	11
+1.20	13	13	13
−0.26	18	18	19
−8.30	51	53	46
−9.80	57	59	51

**Table 3:** *PW3* levels fully in register with *S* levels along the AP axis (*z* mm from Bregma).

***z***	***PW3* Atlas Level**	***S* Atlas Level**
	***PW05, PW07, PW14***	***S92, S98, S04, S18***
+4.20	8	6
+1.20	23	13
0.00	33	17
+0.00	11	11
−1.08	42	23
−4.20	68	33
−13.44	145	67
−14.16	151	70

**Table 4:** Distances between *PW1* & *PW2* levels narrowly in register with *S* levels along the AP axis (*z* mm from Bregma).

***PW*** **Atlas Level**			***S*** **Atlas Level**		**Distance**
***z_*PW*_***	***PW1***	***PW2***	***z_*S*_***	***S92, S98, S04, S18***	**|Δ*z*|, μm**
+6.70	1	1	+6.74	2	40
+2.20	10	10	+2.15	10	50
+1.00	14	14	+0.95	14	50
+0.48	16	16	+0.45	15	30
−0.80	21	21	−0.83	22	30
−1.30	23	23	−1.33	24	30
−1.80	25	26	−1.78	26	20
−2.80	29	31	−2.85	29	50
−3.30	31	33	−3.25	30	50
−4.16	34	36	−4.20	33	40
−4.48	36	38	−4.45	34	30
−5.20	38	40	−5.25	37	50
−5.30	39	41	−5.25	37	50
−5.60	40	42	−5.65	38	50
−6.04	42	44	−6.06	39	20
−6.80	45	47	−6.85	42	50
−7.64	48	50	−7.60	44	40
−8.80	53	55	−8.85	48	50
−10.30	59	61	−10.35	53	50
−10.80	61	63	−10.85	55	50
−11.60	64	66	−11.58	58	20
−11.80	65	67	−11.75	59	50
−12.72	68	70	−12.68	63	40
−13.24	70	72	−13.28	66	40
−13.30	71	73	−13.28	66	20
−13.80	73	75	−13.76	69	40

**Table 5:** Distances between *PW3* levels narrowly in register with *S* levels along the AP axis (*z* mm from Bregma).

***PW*** **Atlas Level**		***S*** **Atlas Level**		**Distance**	***PW*** **Atlas Level**		***S*** **Atlas Level**		**Distance**
***z_*PW*_***	***PW05, PW09, PW14***	***z_*S*_***	***S92, S98, S04, S18***	**|Δ*z*|, μm**	***z_*PW*_***	***PW05, PW09, PW14***	***z_*S*_***	***S92, S98, S04, S18***	**|Δ*z*|, μm**
+5.16	6	+5.20	4	40	−6.60	88	−6.65	41	50
+3.24	10	+3.20	8	40	−6.84	90	−6.85	42	10
+2.76	12	+2.80	9	40	−7.08	92	−7.10	43	20
+2.16	15	+2.15	10	10	−7.56	96	−7.60	44	40
+1.68	19	+1.70	11	20	−7.92	99	−7.90	45	20
+1.44	21	+1.45	12	10	−8.28	102	−8.30	46	20
+0.96	25	+0.95	14	10	−8.64	105	−8.60	47	40
+0.48	29	+0.45	15	30	−8.88	107	−8.85	48	30
+0.12	32	+0.10	16	20	−9.24	110	−9.25	49	10
−0.12	34	−0.11	18	10	−9.48	112	−9.50	50	20
−0.24	35	−0.26	19	20	−9.84	115	−9.80	51	40
−0.48	37	−0.46	20	20	−10.08	117	−10.10	52	20
−0.48	37	−0.51	21	30	−10.32	119	−10.35	53	30
−0.84	40	−0.83	22	10	−10.56	121	−10.60	54	40
−1.32	44	−1.33	24	10	−10.80	123	−10.85	55	50
−1.56	46	−1.53	25	30	−11.76	131	−11.75	59	10
−1.80	48	−1.78	26	20	−11.88	132	−11.90	60	20
−2.04	50	−2.00	27	40	−12.24	135	−12.20	61	40
−2.40	53	−2.45	28	50	−12.48	137	−12.50	62	20
−3.24	60	−3.25	30	10	−12.72	139	−12.68	63	40
−3.72	64	−3.70	31	20	−12.84	140	−12.88	64	40
−4.44	70	−4.45	34	10	−13.20	143	−13.15	65	50
−4.56	71	−4.60	35	40	−13.32	144	−13.28	66	40
−5.04	75	−5.00	36	40	−13.56	146	−13.60	68	40
−5.28	77	−5.25	37	30	−13.80	148	−13.76	69	40
−5.64	80	−5.65	38	10	−14.40	153	−14.36	71	40
−6.48	87	−6.50	40	20	−15.48	159	−15.46	73	20

### Creation and implementation of a computer vision algorithm

In order to provide an independent means to determine whether the external craniometric alignments were a reasonable first-order solution to determine the most similar anteroposterior levels between *PW* and *S* reference spaces, we sought evidence from within the reference tissue sets themselves. This was achieved by developing a computer vision algorithm that enabled feature-based matching of selected regions of interest from within the Nissl-stained tissue used to create each reference space. This tissue is in the form of digital photomicrographs that accompany each atlas edition. For the purposes of algorithm development, test photomicrographs were used from the seventh edition of *PW* (Paxinos and Watson, 2014) and the third edition of *S* (Swanson, 2004).

#### Basic operations of the custom-made computer vision algorithm

**Figure 3:**
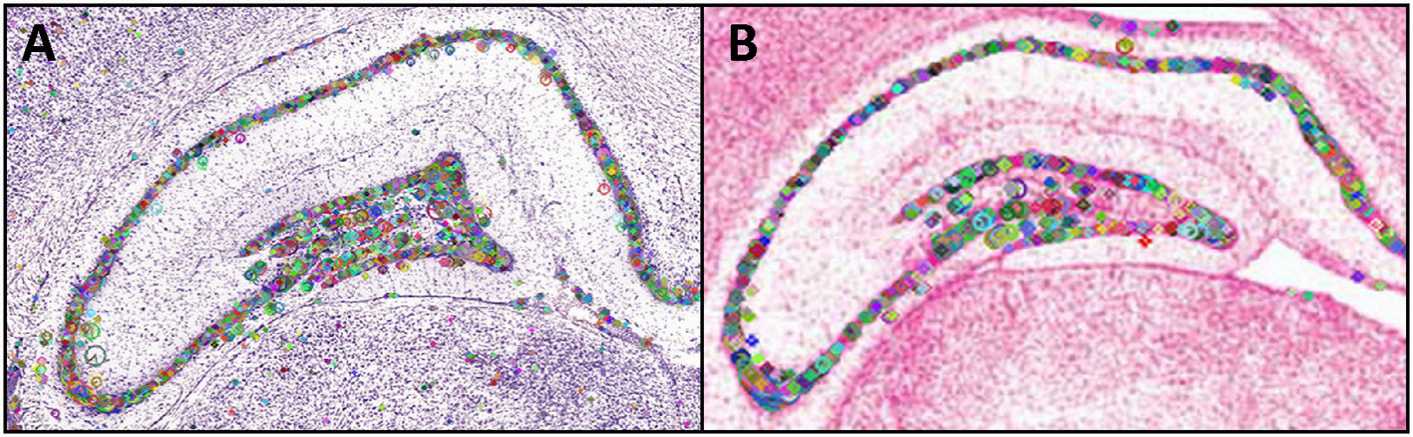
Points of interest found in a region of interest from a plate in the **(A)** Paxinos and Watson (2014) atlas and **(B)** in the Swanson (2004) atlas. The small horizontal lines in each panel inside the circular regions indicate the region's dominant orientation. Different region sizes correspond to different scales. The portion of the *PW14* atlas photomicrograph (Level 70) is reproduced in **(A)** with permission from Elsevier. The photomicrograph in **(B)** is reproduced from Swanson (2004) under the conditions set forth by a Creative Commons BY-NC 4.0 license (https://creativecommons.org/licenses/by-nc/4.0/legalcode).

Figure 3 shows the points of interest found in sampled parts of Nissl-stained photomicrographs from Paxinos and Watson (2014) (Figure 3A) and from Swanson (2004) (Figure 3B). The small lines inside the circles each indicate the dominant orientation of the area, with different region sizes corresponding to different scales.

#### Results of *Experiments 1a* and *1b*: feature-based matching to tissue section of origin

**Figure 4:**
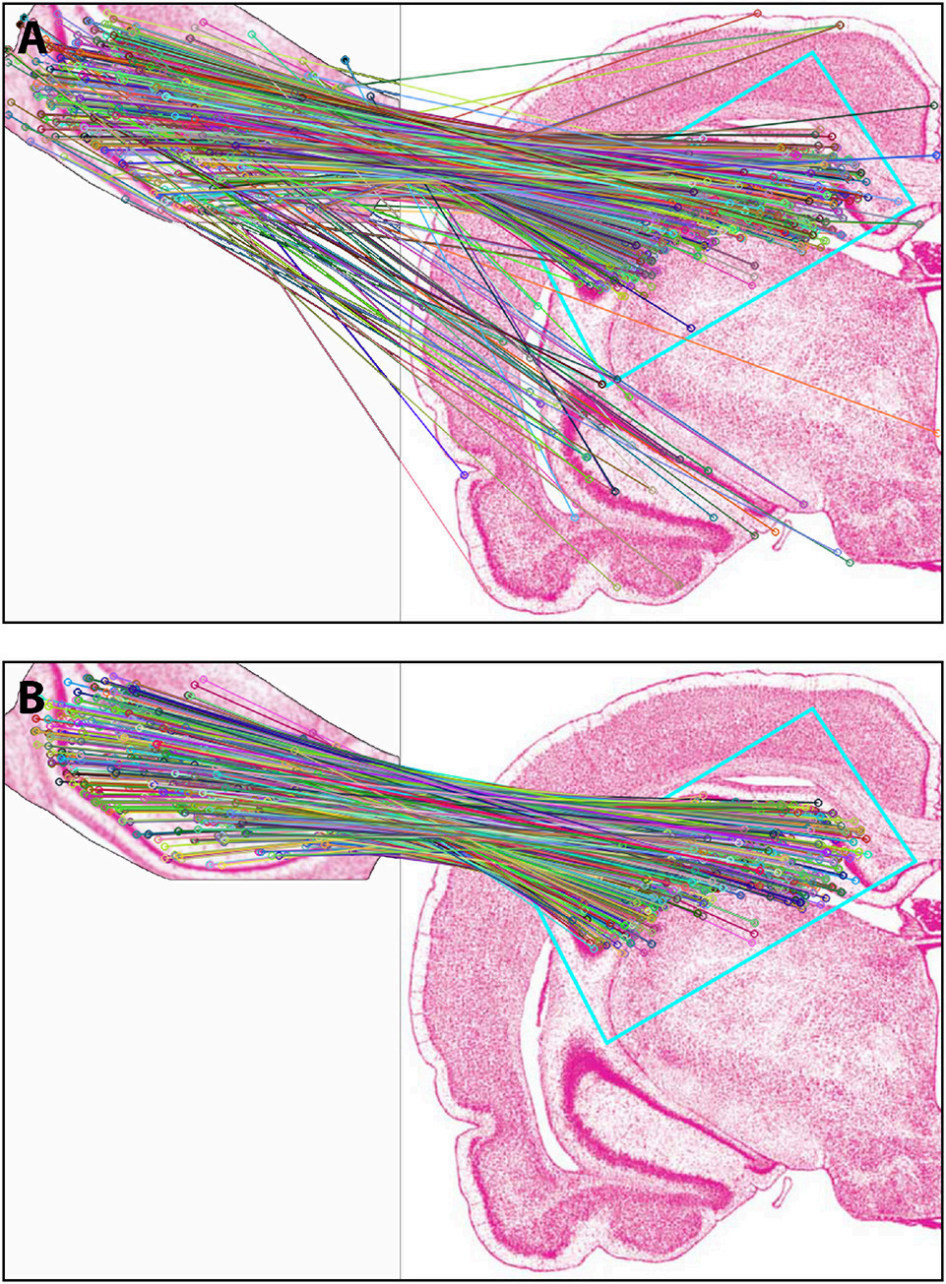
Results of *Experiment 1a*. The algorithm successfully matched the exact *S* atlas plate (Level 34), containing the desired Nissl-stained tissue section, with the ROI extracted digitally from that section, which was rotated 155 degrees and distorted slightly through random point-warping, and used as a test image. SIFT matches are shown for the test image before **(A)** and after **(B)** RANSAC was applied to remove outliers. The photomicrographs are reproduced from Swanson (2004) under the conditions set forth by a Creative Commons BY-NC 4.0 license (https://creativecommons.org/licenses/by-nc/ 4.0/legalcode).

**Figure 5:**
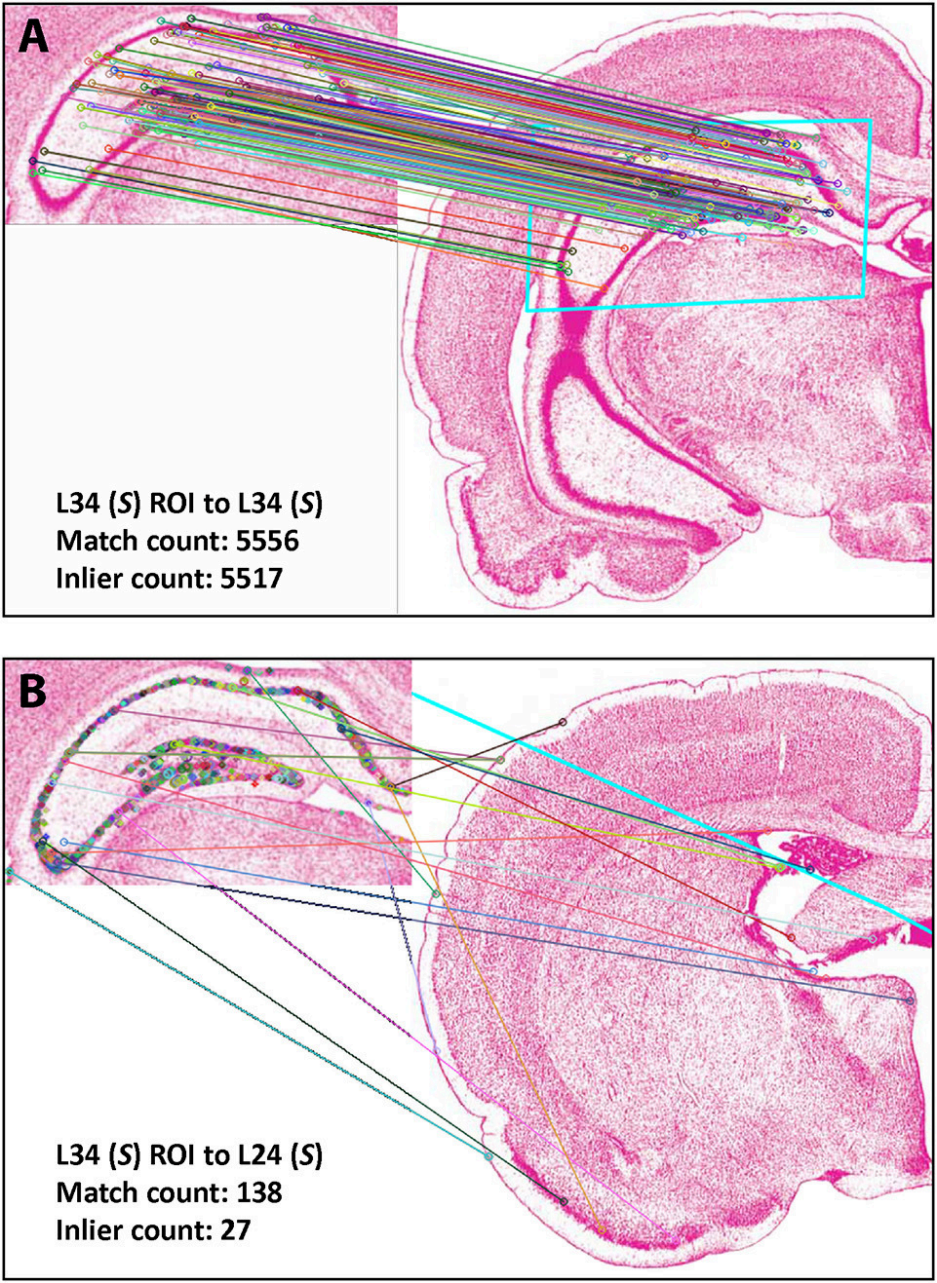
Results of *Experiment 1b*. Examples of a good match between an ROI and an image **(A)**, and a poor one **(B)**. The photomicrographs are reproduced from Swanson (2004) under the conditions set forth by a Creative Commons BY-NC 4.0 license (https://creativecommons.org/licenses/by-nc/4.0/legalcode).

Figure 4 shows the results of *Experiment 1a*. A region of interest was extracted from the Nissl image corresponding to Level 34 of *S* space, rotated and distorted, and then used as a test ROI for the algorithm to produce the correct *S* level from which the test region originated. The results of SIFT operations before and after the application of RANSAC are shown (Figures 4A,B, respectively). Figure 5 and Table 6 show representative results of *Experiment 1b*, comparing the feature-based matching of an ROI from Level 34 of *S* to its correct and incorrect tissue photomicrograph of origin from the *S* atlas. As shown in Figure 5A, the algorithm successfully matched a number of features of the ROI to the plate of origin. The highest number of SIFT matches and RANSAC inliers was for Level 34, with SIFT matches being nearly three times greater and RANSAC inliers an order of magnitude greater, respectively, than the values of the next highest ranking plate match (Table 6). In contrast, images that clearly were from a different *S* plate, such as Level 22 shown in Figure 5B, resulted in a predictably low number of matches and inliers.

**Table 6:** Top matches (Experiments 1b & 2).

***S* Level**	**SIFT Matches**	**RANSAC Inliers**
**EXPERIMENT 1b**		
34	3618	3494
71	1316	822
35	652	278
**EXPERIMENT 2**		
30	483	212
29	456	197
32	431	173
34	410	144
36	525	137

#### Results of *Experiment 2*: feature-based matching to determine plate correspondence

**Figure 6:**
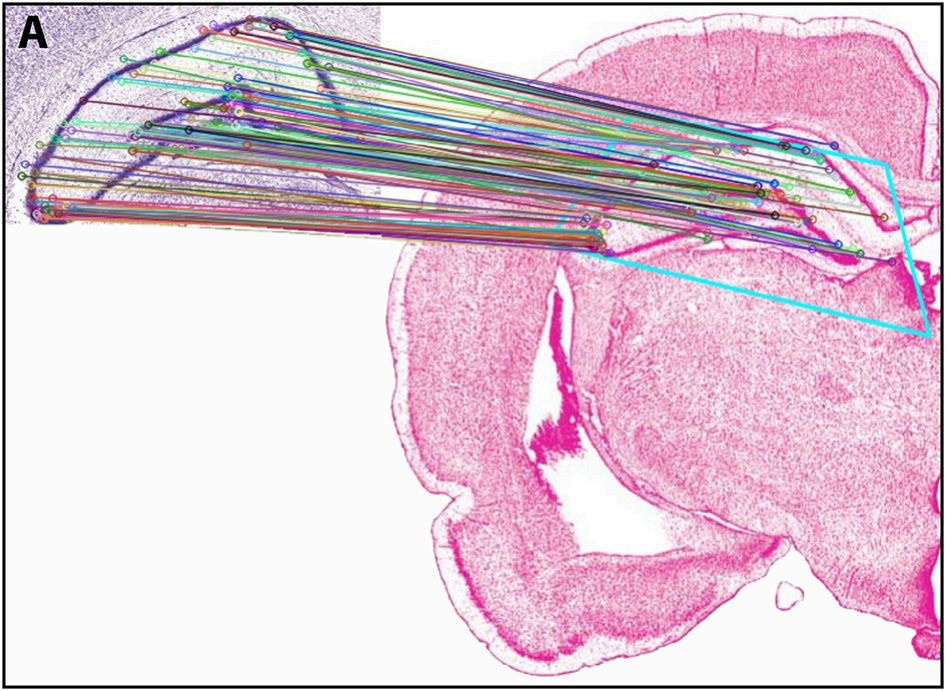
Results of *Experiment 2*. View of the match obtained after *Experiment 2* was implemented, between an ROI extracted from *PW* space and the closest matching *S* atlas photomicrograph. In the inset (labeled A), the portion of the *PW14* atlas photomicrograph (Level 70) is reproduced with permission from Elsevier. The photomicrograph in the main image is reproduced from Swanson (2004) under the conditions set forth by a Creative Commons BY-NC 4.0 license (https://creativecommons.org/licenses/by-nc/4.0/legalcode).

**Figure 7:**
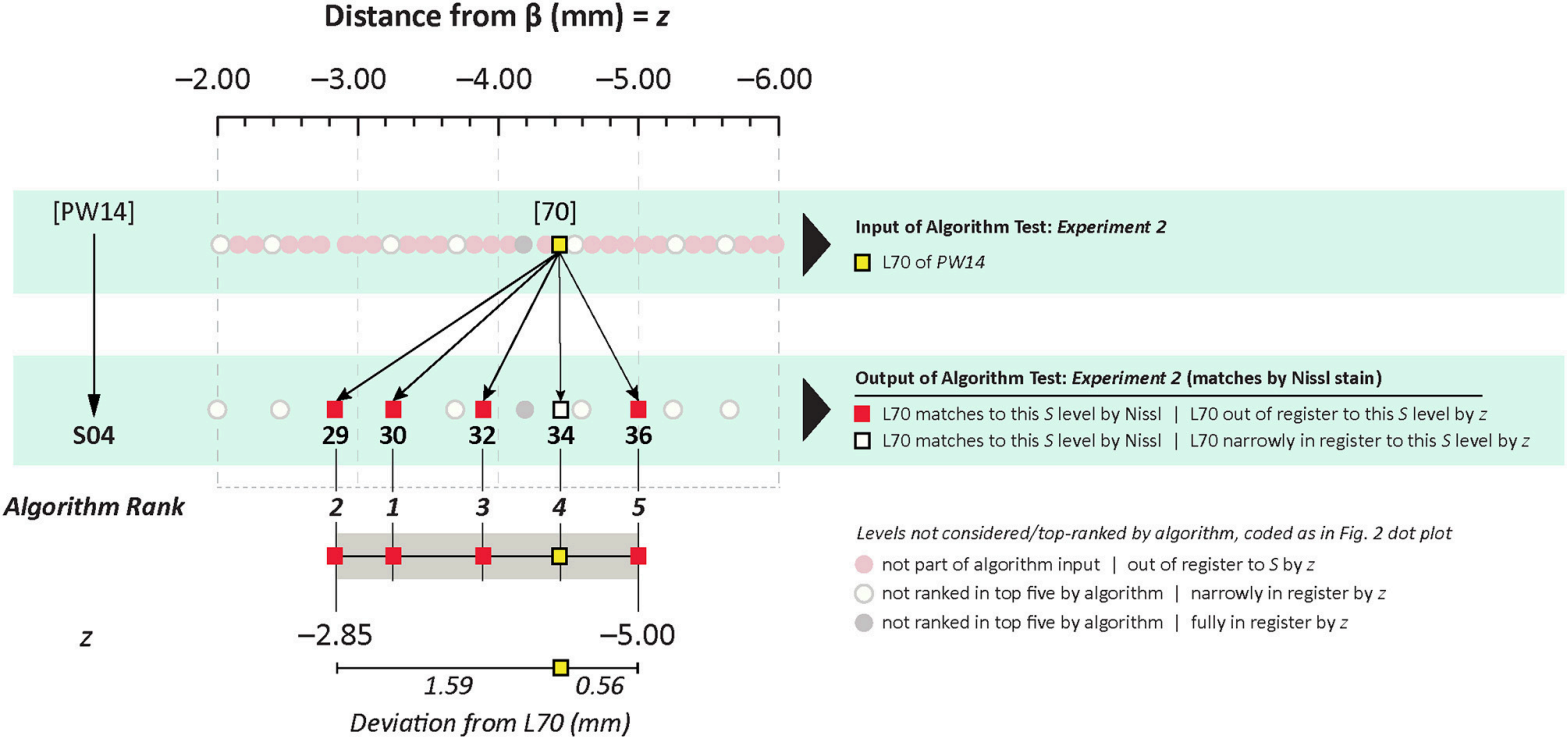
Summary of *Experiment 2*. The algorithm successfully matched its top-ranked *S* atlas plates (most plates within Levels 29–36) with the ROI extracted digitally from Plate 70 of *PW3* space. This range is in close agreement with the general range of levels between the two reference spaces, as indicated by craniometric measures.

In *Experiment 2*, an ROI from the Nissl image associated with a specific plate in *PW* reference space (Plate 70 (L70) of *PW14*) was used as a test for the algorithm's ability to determine the appropriately matched Nissl plate in *S* space. Figure 6 shows the robust feature-based matching that the algorithm achieved for the top-ranked *S* photomicrographic plate. Table 6 shows the values for both the SIFT matches and RANSAC inliers for the five highest-ranking matches, which are in the range of *S* Levels 29–36. As can be seen in Table 1 and Figure 7, the levels within this range are all flanking *S* L34, which is assigned as being *narrowly in register* with *PW* L70 on the basis of craniometric alignments (Figure 2B). These results support craniometric alignments as being a reliable first-order means to align the two reference spaces along the anteroposterior axis.

### Data transformation

**Figure 8:**
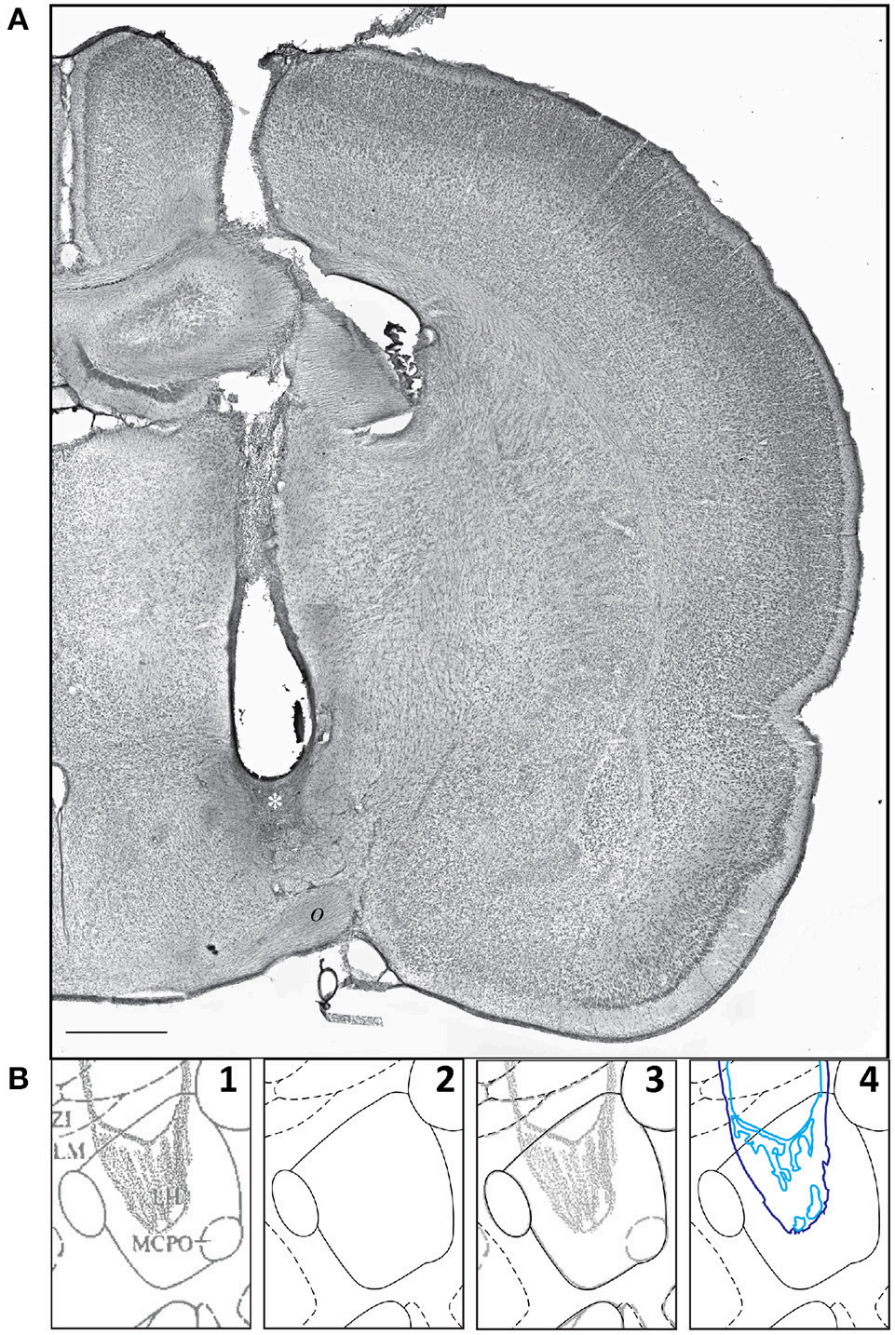
**(A)** A representative example of a microinjection site, with the injection scar denoted by a white asterisk (*). This photomicrograph shows one half of a transverse section through the rat brain, with a needle track targeting the hypothalamus. *o*, optic tract. Scale bar = 1 mm. **(B)** Sequential steps in the transformation of a graphical injection site drawing to a vector-formatted object: *Step 1*: graphical drawing based on *PW*86; *Step 2*: the *PW*98 digital version of the same atlas drawing; *Step 3*: an overlay of the drawing in 1 and the digital drawing in 2 (graphical drawing over digital boundaries); *Step 4*: vector drawing, in a separate layer, of the injection site. The portion of the *PW* atlas figure (Level 26) is reproduced here with permission from Elsevier.

Khan et al. (2004) reported the effects of centrally microinjecting membrane-permeable protein tyrosine kinase inhibitors (PTKIs) on feeding behavior produced by central injections of the glutamate receptor agonist, *N*-methyl-d-aspartate (NMDA). Specifically, the PTKIs, Tyrphostin A48 and PP1, were able to powerfully suppress NMDA-elicited eating when injected into the lateral hypothalamic area. However, in their study, the central microinjection sites were not published. We therefore decided to use these unpublished injection sites as a test for our data migration procedures and to publish their locations in both *PW* and *S* reference spaces. The first step needed to enable data migration for these sites was their transformation from graphical to digital form, and then their conversion from raster to vector format within digital space. Figure 8 shows a representative example of a central microinjection site (Figure 8A) and the steps by which its drawn 2-D representation was transferred into a digital vector format (Figure 8B, Panels 1–4).

### Data migration

#### Migration

**Figure 9:**
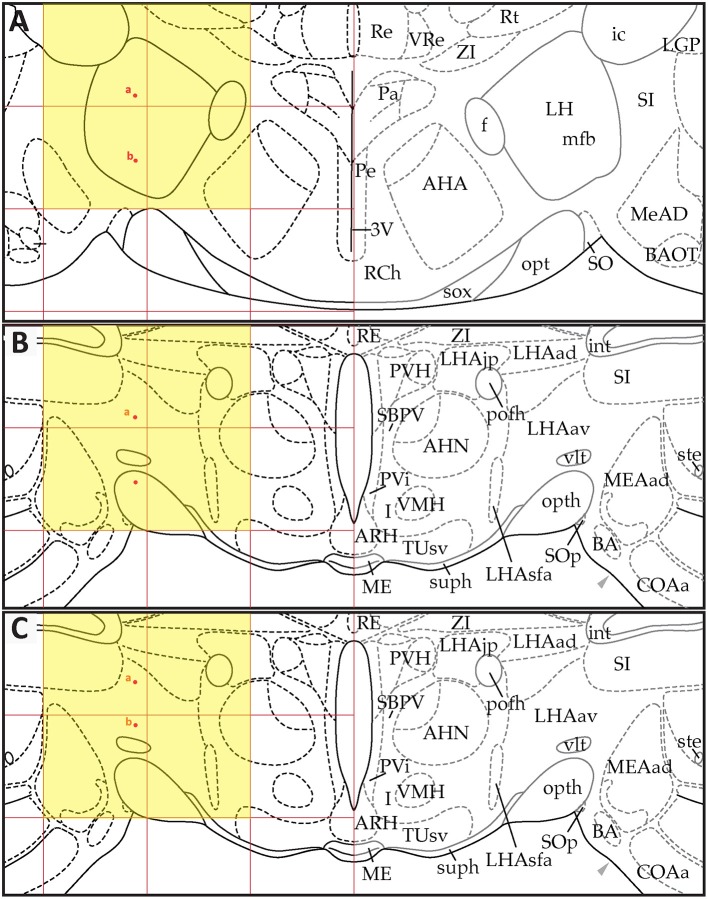
Point-source data migrated from the digital map in Paxinos and Watson (1998), Figure 26, to Swanson (2018), Level 26. **(A)** Paxinos and Watson (1998), Figure 26. Point-source data are shown by red dots, which have been shifted contralaterally. **(B)** Swanson (2018), Level 26, with stereotaxic grid aligned to that of Paxinos and Watson (1998), Figure 26. Point-source data appear at their original stereotaxic coordinates. **(C)** Swanson (2018), Level 26, with stereotaxic grid aligned to that of Paxinos and Watson (1998), Figure 26. Point-source data have been shifted to more closely match their original locations with regard to nearby fiducials. Note that to minimize reader distraction, the general appearance (but not boundaries or nomenclature) of the *S* levels in **(B**,**C)** have been altered to match those of *PW*. Figure 26 from *PW98* is reproduced with permission from Elsevier. Level 26 from *S18* is reproduced from Swanson (2018) under the conditions of a Creative Commons BY-NC 4.0 license (https://creativecommons.org/licenses/by-nc/4.0/legalcode). For an explanation of the abbreviations in **A**, see List of Abbreviations, Paxinos and Watson Nomenclature. For an explanation of the abbreviations in **B** and **C**, please see List of Abbreviations, Swanson Nomenclature.

**Figure 10:**
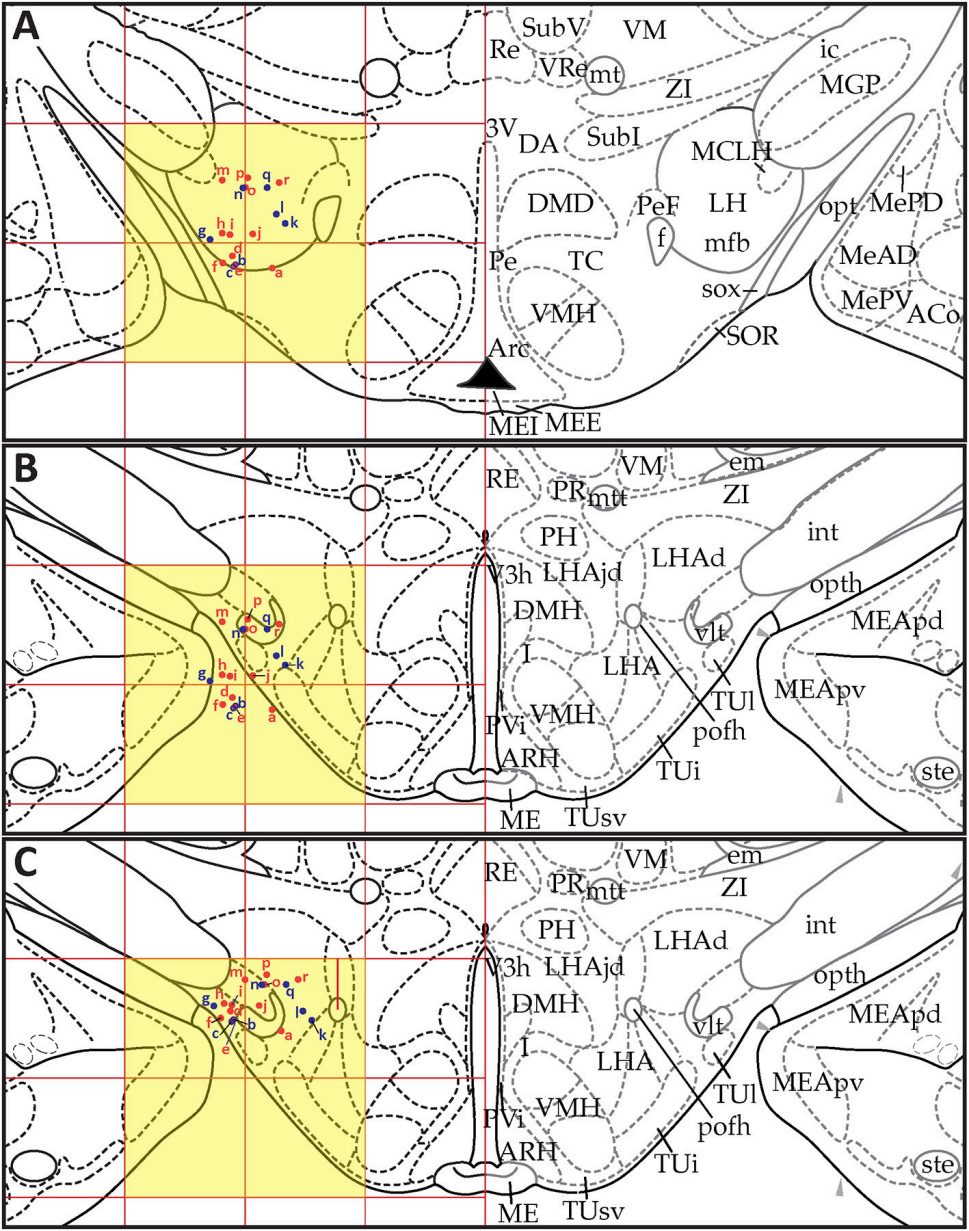
Point-source data migrated from Paxinos and Watson (1998), Figure 31, to Swanson (2018), Level 29. **(A)** Paxinos and Watson (1998), Figure 31. Point-source data are shown by red dots, which have been shifted contralaterally; and blue dots, which have not been shifted. **(B)** Swanson (2018), Level 29, with stereotaxic grid aligned to that of Paxinos and Watson (1998), Figure 31. Point-source data appear at their original stereotaxic coordinates. **(C)** Swanson (2018), Level 29, with stereotaxic grid aligned to that of Paxinos and Watson (1998), Figure 31. Point-source data have been shifted to more closely match their original locations with regard to nearby fiducials. Note that to minimize reader distraction, the general appearance (but not boundaries or nomenclature) of the *S* levels in **(B,C)** have been altered to match those of *PW*. Figure 31 from *PW98* is reproduced with permission from Elsevier. Level 29 from *S18* is reproduced from Swanson (2018) under the conditions of a Creative Commons BY-NC 4.0 license (https://creativecommons.org/licenses/by-nc/4.0/legalcode). For an explanation of the abbreviations in **A**, see List of Abbreviations, Paxinos and Watson Nomenclature. For an explanation of the abbreviations in **B** and **C**, please see List of Abbreviations, Swanson Nomenclature.

**Figure 11:**
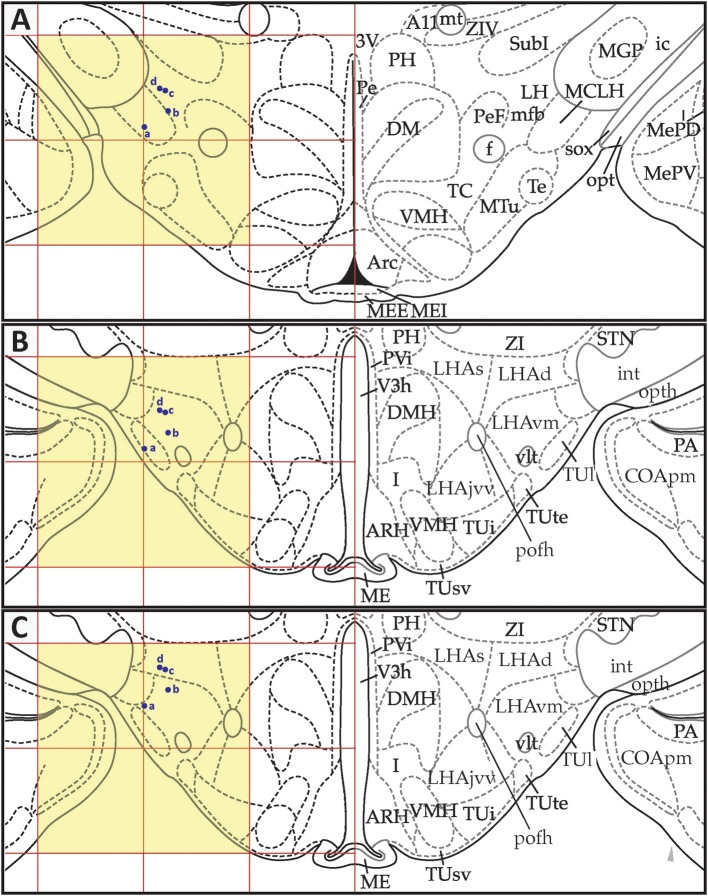
Point-source data migrated from Paxinos and Watson (1998), Figure 33, to Swanson (2018), Level 30. **(A)** Paxinos and Watson (1998), Figure 33. Point-source data are shown by blue dots. **(B)** Swanson (2018), Level 30, with stereotaxic grid aligned to that of Paxinos and Watson (1998), Figure 33. Point source information appears at its original stereotaxic coordinates. **(C)** Swanson (2018), Level 30, with stereotaxic grid aligned to that of Paxinos and Watson (1998), Figure 33. Point source data have been shifted to more closely match their original locations with regard to nearby fiducials. Note that to minimize reader distraction, the general appearance (but not boundaries or nomenclature) of the *S* levels in **(B**,**C)** have been altered to match those of *PW*. Figure 33 from *PW98* is reproduced with permission from Elsevier. Level 30 from *S18* is reproduced from Swanson (2018) under the conditions of a Creative Commons BY-NC 4.0 license (https://creativecommons.org/licenses/by-nc/4.0/legalcode). For an explanation of the abbreviations in **A**, see List of Abbreviations, Paxinos and Watson Nomenclature. For an explanation of the abbreviations in **B** and **C**, please see List of Abbreviations, Swanson Nomenclature.

The alignment tool in Figure 2A was used to determine the atlas level in *S* space that corresponds most closely to the *PW* level serving as the source of the migrated injection site data. The *PW* and *S* reference spaces were first brought into register with one another in the mediolateral and dorsoventral axes. This was achieved by taking advantage of the stereotaxic grid embedded in the .ai files comprising the digital atlas maps of Swanson (2004), which was provided to users of the atlas as a means to contextualize their data according to the stereotaxic coordinates of Paxinos and Watson (1986). Using this grid and the scaling factors provided by Swanson (1992), three atlas maps from *PW*98 were aligned with the corresponding closest matching maps in *S*04: *PW*98_26_
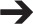
*S*_26_, *PW*98_31_
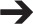
*S*_29_, and *PW*98_33_
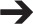
*S*_30_. A point-source datum representing the ventral tip of each injection site was drawn on the 2-D rendering of the injection site, and each of these data points was then migrated to the appropriate *S* level. Figures 9–**11** show the datasets for the *PW*98_26_
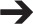
*S*_26_, *PW*98_31_
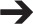
*S*_29_, and *PW*98_33_
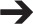
*S*_30_ migrations, respectively. Each of these figures shows three panels (A–C) that depict the positions of the data points in *PW*98 space (Figures 9A, 10A, 11A), their transfer to *S* space (Figures 9B, 10B, 11B), and finally, their adjusted positions in *S* space after expert-guided corrections to the migrated datasets were performed (Figures 9C, 10C, 11C).

#### Analysis

**Figure 12:**
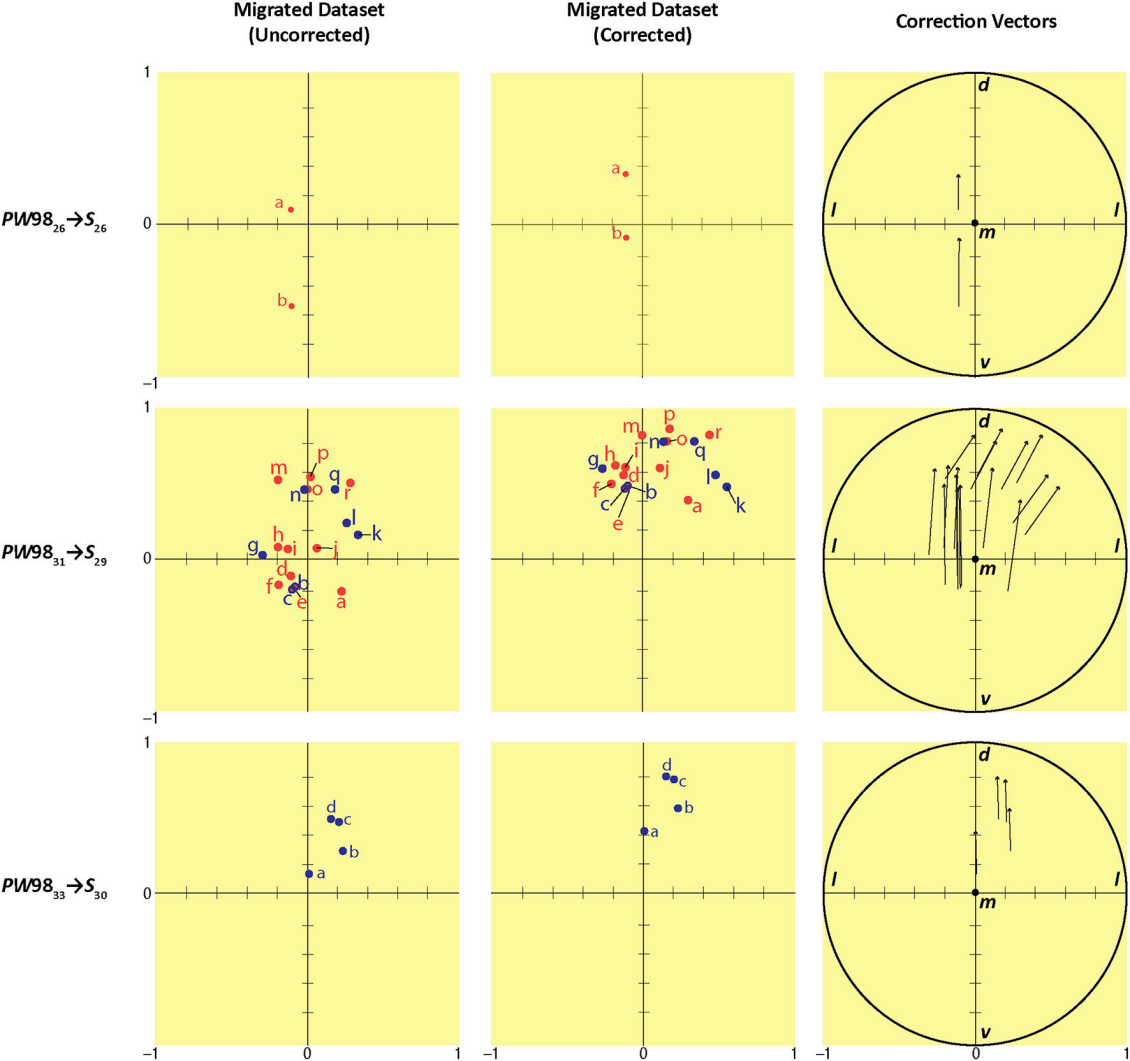
Summary of migration analysis conducted by converting each *S* atlas reference quadrant into a Cartesian plane. Scales on the ordinate and abscissa are in millimeter units. Each row shows the migration from a different source *PW* level to its corresponding (most closely matching) level in *S* space: *PW*98_26_
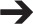
*S*_26_ (*top row*), *PW*98_31_
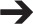
*S*_29_ (*middle row*), and *PW*98_33_
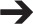
*S*_30_ (*bottom row*). The columns show the position of the migrated points in *S* space before (*left column*) and after (*middle column*) expert-guided mapping adjustments and corrections were made. *Right column*: the vectors for each pair of original and relocated points are shown within a unit circle with origin *m* (to denote the medial-most point), mediolateral *x*-axis (*l*, lateral), and dorsoventral *y*-axis (*d*, dorsal; *v*, ventral). Point-source data are shown by red dots, which have been shifted contralaterally; and blue dots, which have not been shifted.

In order to ascertain the amount of error in data migration that required correction by a subject matter expert, we quantitatively determined the location of each migrated data point within a Cartesian workspace derived from the original reference space quadrants into which the data were migrated (*S* space from *PW* space) (Figure 12). As seen in the *left column* of data points in Figure 12, the positions of all data points fell within a 2 × 2 mm Cartesian plane, as did the locations of the relocated points after expert intervention (Figure 12, *middle column*). The differences in positions for the original vs. relocated data points were expressed in the form of vectors (Figure 12, *right column*). The magnitude and direction of each vector were calculated as described in the Methods. The results of these calculations are summarized in Table 7. The average magnitude of the vectors was 76 μm in the mediolateral dimension and 442 μm in the dorsoventral dimension (Table 7), demonstrating that the error in migrating the data was dominated by errors in the latter dimension. This was supported by the arctangent calculations computed for each vector, which produced a mean value of 78.4° for φ. If φ had been less than 45°, the result would have suggested a more dominant deviation laterally (i.e., along the ML axis), but this was not the case. Thus, both the mean *xy* components of the vector and its mean direction demonstrate that the overall correction performed by the expert user was to shift the migrated points in the dorsolateral direction, with the greater component of this correction occurring along the DV axis.

**Table 7:** Positions, errors, and corrections for migrated datasets.

**Levels**	**Subject # (Expt #)**	**Label**	**Distance of Scaled Data from Cartesian Origin (mm)**				**Expert-Guided Mapping**			
			− **Expert guidance**		+ **Expert guidance**		**Error (mm)**		**Correction**	
			**A*_*x*_* (ML)**	**A*_*y*_* (DV)**	**B*_*x*_* (ML)**	**B*_*y*_* (DV)**	**AB*_*x*_* (ML)**	**AB*_*y*_* (DV)**	**AB**	**φ°**
*PW*_26_ 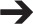 *S*_26_	00/115 (5b)	a	−0.12776	−0.11052	−0.12456	−0.34712	0.00320	0.23660	0.236622	89.225
	00/123 (5b)	b	−0.12360	0.53560	−0.12040	0.07792	0.00320	0.45768	0.457691	89.599
*PW*_31_ 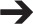 *S*_29_	99/153 (4)	a	0.20612	0.16380	0.29084	−0.41396	0.08472	0.57776	0.583938	81.658
	99/144 (4)	b	−0.10444	0.16428	−0.11252	−0.51160	0.00808	0.67588	0.675928	89.315
	99/152 (4)	c	−0.12844	0.18468	−0.13288	−0.49116	0.00444	0.67584	0.675855	89.624
	99/138 (4)	d	−0.13240	0.09252	−0.14044	−0.58232	0.00804	0.67584	0.675888	89.318
	00/049 (5a)	e	−0.11320	0.17572	−0.12124	−0.50012	0.00804	0.67584	0.675888	89.318
	00/036 (5a)	f	−0.21412	0.15276	−0.22216	−0.52308	0.00804	0.67584	0.675888	89.318
	99/151 (4)	g	−0.32236	−0.04632	−0.28208	−0.62544	0.04028	0.57912	0.580519	86.021
	00/044 (5a)	h	−0.21908	−0.09960	−0.19492	−0.64652	0.02416	0.54692	0.547453	87.471
	00/092 (*)	i	−0.15284	−0.08728	−0.12868	−0.63420	0.02416	0.54692	0.547453	87.471
	00/039 (5a)	j	0.04104	−0.09264	0.10288	−0.62924	0.06184	0.53660	0.540152	83.426
	99/143 (4)	k	0.31640	−0.18180	0.55008	−0.50312	0.23368	0.32132	0.397307	53.973
	99/137 (4)	l	0.24096	−0.26124	0.47464	−0.58256	0.23368	0.32132	0.397307	53.973
	00/057 (*)	m	−0.21908	−0.54960	−0.01672	−0.84860	0.20236	0.29900	0.361041	55.910
	99/147 (4)	n	−0.04248	0.48416	0.12672	−0.80544	0.16920	0.32128	0.363111	62.227
	00/084 (*)	o	−0.02108	−0.48688	0.14808	−0.80820	0.16916	0.32132	0.363128	62.235
	00/050 (5a)	p	−0.00176	−0.56996	0.16744	−0.89124	0.16920	0.32128	0.363111	62.227
	99/135 (4)	q	0.16320	−0.56996	0.33240	−0.80776	0.16920	0.32128	0.363111	62.227
	00/086 (*)	r	0.26500	−0.52916	0.43420	−0.85048	0.16920	0.32132	0.363146	62.230
*PW*_33_ 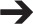 *S*_30_	99/153 (4)	a	−0.01084	−0.14860	−0.01832	−0.44964	0.00748	0.30104	0.301133	88.577
	99/144 (4)	b	0.21572	−0.30200	0.20692	−0.60216	0.00880	0.30016	0.300289	88.321
	99/152 (4)	c	0.18920	−0.49628	0.18056	−0.79532	0.00864	0.29904	0.299165	88.345
	99/138 (4)	d	0.13552	−0.51648	0.12720	−0.81544	0.00832	0.29896	0.299076	88.406
	Mean		−0.00668	−0.16578	0.06363	−0.60778	0.07613	0.44201	0.460175	78.351
	SEM		0.03564	0.06012	0.04593	0.04195	0.01679	0.03134	0.028963	2.747

* *indicates an experiment conducted in association with those published in Khan et al. (2004) but not included in that study. Letter designations in the “Label” column refer to labels in Figures 9–11 which mark the locations for each injection site*.

#### Data decoding

**Figure 13:**
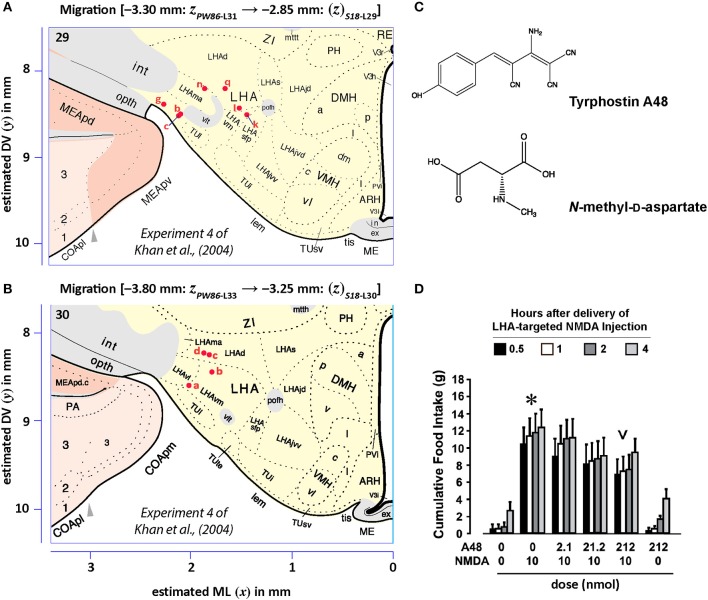
Migrated data coded by behavioral experiment. The injection cases involving *Experiment 4* of Khan et al. (2004) are shown in their final migrated states. In **(A)**, the injection sites migrated from Paxinos and Watson (1986) (*PW86*), Level 31, are shown in their host reference space [(Swanson, 2018) (*S18*); Level 29]. In **(B)** the injection sites migrated from *PW86*, Level 33, are shown in *S18*, Level 30. The scales flanking these panels mark the estimated stereotaxic coordinates in the mediolateral (*x*) and dorsoventral (*y*) dimensions, derived from *PW86* and encoded within *S18*. The *x*-axis scale applies to both **(A,B)**. Note that expert-guided intervention was required to make adjustments of the injection sites to their final locations, and thus the plotted points should be considered as first-order approximations of the actual injection sites. The letters denoting each site refer to case numbers, which are found for the corresponding levels in Table 7 for the “*PW*_31_
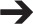
*S*_29_” migration **(A)** and “*PW*_33_
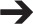
*S*_30_” **(B)**. **(C)** Structures of the reagents injected: the protein tyrosine kinase inhibitor, Tyrphostin A48 (A48), and the glutamate receptor agonist, *N*-methyl-d-aspartate (NMDA). **(D)** The behavioral results, adapted from Khan et al. (2004), associated with the injection sites mapped in A and B. Two injections were delivered to each site, 10 min apart, with A48 injected before NMDA. All injection volumes were 300 nl, containing the doses of the reagents as indicated. The asterisk marks significant overall cumulative food intake triggered by NMDA injection relative to vehicle injection, and the inverted carat denotes significant suppression of NMDA-elicited eating at the highest dose of A48 tested (*P* < 0.5). See Khan et al. (2004) for details. Permission to reproduce the data in **D** from Khan et al. (2004) has been provided under the permissions policy of *The Journal of Neuroscience*. Levels 29 and 30 from *S18* are reproduced from Swanson (2018) under the conditions of a Creative Commons BY-NC 4.0 license (https://creativecommons.org/licenses/by-nc/4.0/legalcode). For an explanation of the abbreviations on this figure, please see the List of Abbreviations, Swanson Nomenclature.

**Figure 14:**
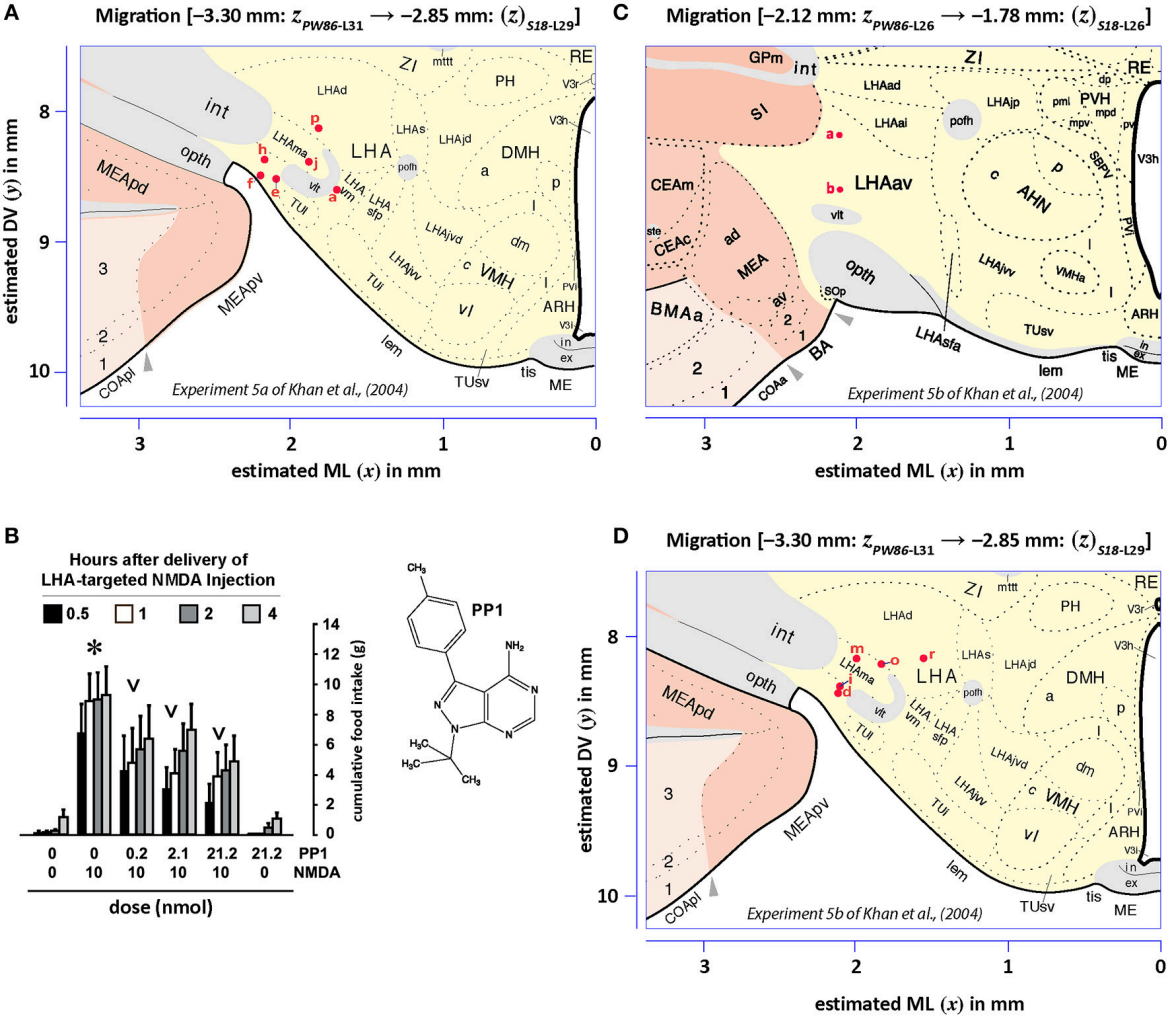
Migrated data coded by behavioral experiment. The injection cases involving *Experiment 5a* and *Experiment 5b* of Khan et al. (2004) are shown in their final migrated states. In **(A)**, the injection sites for *Experiment 5a*, migrated from Paxinos and Watson (1986) (*PW86*), Level 31, are shown in their host reference space (Swanson, 2018 (*S18*); Level 29). In **(B)**, the behavioral data, adapted from Khan et al. (2004), are shown that are associated with the injection sites in **(A)**, along with the chemical structure of the protein tyrosine kinase inhibitor, PP1. Two injections were delivered to each site, 10 min apart, with PP1 injected before NMDA. All injection volumes were 300 nl, containing the doses of the reagents as indicated. The asterisk denotes significantly greater cumulative food intake overall relative to vehicle controls, and the inverted carats denote significant suppression of cumulative food intake relative to NMDA-elicited eating (*P* < 0.5). See Khan et al. (2004) for details. In **(C,D)**, the injection sites for *Experiment 5b*—and for an associated experiment not reported in Khan et al. (2004) (see cases marked with an asterisk in Table 7)—migrated from *PW86*, Level 26 and 31; are shown in *S18*, Level 26 and 29, respectively. Associated behavioral results for this experiment are not shown here, but can be found in the descriptive narrative of the Results section in Khan et al. (2004). The scales flanking these panels mark the estimated stereotaxic coordinates in the mediolateral (*x*) and dorsoventral (*y*) dimensions, derived from *PW86* and encoded within *S18*. The *x*-axis scale in **(A)** applies to both **(A,B)**. Note that expert-guided intervention was required to make adjustments of the injection sites to their final locations, and thus the plotted points should be considered as first-order approximations of the actual injection sites. The letters denoting each site refer to case numbers, which are found for the corresponding levels in Table 7 for the “*PW*_31_
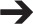
*S*_29_” migration **(A)** and “*PW*_26_
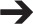
*S*_26_” and “*PW*_31_
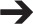
*S*_29_” migrations **(C,D)**. Permission to reproduce the data in **B** from Khan et al. (2004) has been provided under the permissions policy of *The Journal of Neuroscience*. Levels 26 and 29 from *S18* are reproduced from Swanson (2018) under the conditions of a Creative Commons BY-NC 4.0 license (https://creativecommons.org/licenses/by-nc/4.0/legalcode). For an explanation of the abbreviations on this figure, please see the List of Abbreviations, Swanson Nomenclature.

Figures 13, 14 show summaries of the migrated data points from Figures 9–11, but decoded with respect to the experiments conducted in the original study (Khan et al., 2004). Specifically, three different experiments were performed using protein tyrosine kinase inhibitors (PTKIs). In the first experiment (Exp. 4 in Khan et al., 2004), the PTKI, Tyrphostin A48, was tested at varying doses against NMDA (*TyrA48 dose-response*). In the second experiment (Exp. 5a in Khan et al., 2004), PP1 was tested at varying doses against NMDA (*PP1 dose-response*). Finally, in a third experiment (Exp. 5b in Khan et al., 2004), two doses of PP1 were again tested against the feeding stimulatory effects of NMDA (*PP1* vs. *NMDA*). Figures 13, 14 show the injection sites, sorted and then migrated onto Swanson (2018) atlas maps, for each of these three coded experiments, along with a few injection sites from a related experiment involving PP1 that was not reported in Khan et al. (2004) (see Table 7). In the host *S18* reference space, the injection sites fell within the following portions of the lateral hypothalamic area: anterior group, anterior region, ventral zone (LHAav; Figure 14C); middle group, lateral tier, dorsal region (LHAd; Figures 13A,B, 14A,D); middle group, lateral tier, ventral region, magnocellular nucleus (LHAma; Figures 13A, 14A,D); and middle group, lateral tier, ventral region, medial zone (LHAvm; Figures 13A, 14A). Several injection sites were also migrated to a region near but not within the lateral hypothalamic area, middle group, lateral tier, tuberal nucleus, lateral part (TUl; Figures 13A, 14A).

## Discussion

In this study, we sought to establish a basic framework for migrating spatial datasets between two sets of canonical reference spaces for the rat brain, the Paxinos & Watson (*PW*) and Swanson (*S*) rat brain atlases. The major findings from this effort can be summarized as follows. First, concerning *alignment*, calibrating the *PW* and *S* atlas levels to the Bregma landmark allowed a basic tool to be created to interrelate the reference spaces. Second, concerning *matching*, the novel computer vision algorithm we created to match the image features from atlas photomicrographs of Nissl-stained tissue provided independent support for the utility and general accuracy of the craniometric alignments. Finally, with regard to *migration*, we demonstrate that the transfer of unpublished spatial datasets from a behavioral study of the hypothalamus between rostrocaudally registered *PW* and *S* spaces could be achieved by expert-guided mapping in the mediolateral and dorsoventral dimensions. Each of these outcomes will be discussed below in relation to the value of data migration across atlases for domain experts in behavioral neuroscience and neuroanatomy.

### Craniometric alignment

The approach taken in this study to interrelate the *PW* and *S* reference spaces owes its existence, in part, to the prescient decision made by Swanson (1992) to provide a detailed description of the spatial relationships between his atlas and that published by Paxinos and Watson (1986). The first attempt to interrelate the two reference spaces, therefore, occurs in Swanson (1992); our approach should be considered as simply an independent attempt to do the same. Additionally, from the basic starting point set down by Swanson (1992), we have taken the logical next step of applying and testing his basic system of *PW*/*S* registration across each of the levels that collectively populate the other editions of *PW* and *S* that have been published since 1992.

The rationale for performing such registration is based on certain favorable starting conditions that suggest immediately to discerning users of both series of atlases that registration between the two would appear to be feasible. First, the plane of section for the brain used to create the *S* atlas series, at least through the rostral forebrain and midbrain, is very similar to that provided by Paxinos and Watson (1986), reportedly differing by only four degrees in the mediolateral plane (Swanson, 1992). Second, *S* space derives its stereotaxic coordinates from the *PW*86 atlas, and scaling factors in both mediolateral and dorsoventral dimensions have been provided in Swanson (1992) to facilitate registration. Finally, the vector graphics files provided by Swanson (1992) in Adobe Illustrator format, together with AI's native system of data layers, provide a very useful means to scale the maps from both atlas series using transparent overlays.

The alignment tool we furnish in this study has been created with the needs of the behavioral neuroscientist in mind. We selected 50 μm as the interval that defines levels between *PW* and *S* reference spaces that are *narrowly in register* vs. *not in register* with one another, because this interval is greater in resolution than the resolution of most probes used for manipulations in the rat, which typically range from 100 to 300 μm in diameter (Zhang et al., 2010; Larson et al., 2015; Gigante et al., 2016). In our experience (e.g., Khan et al., 1999, 2004, 2007), greater resolution is not required at this time to achieve the practical goals of performing a successful, reproducible intracranial manipulation of neural substrates in this animal model. Higher resolution may, in fact, impair the clarity required of any useful map for a bench neuroscientist (Wagner, 2011). Of course, as the technology used to manipulate neural substrates becomes smaller in scale (e.g., Kim et al., 2013), the need for further refinements in resolution will arise for maps to remain maximally useful.

### Image feature detection and matching

Despite the parameters described in the preceding section that suggest the feasibility of bringing *S* and *PW* spaces into register with one another, we sought an independent means to determine whether such registration was accurate. This is because the use of Bregma coordinates has been evaluated in the context of registration of rat brain representations by others, and has been reported to be error-prone (Kline and Reid, 1984; Santori and Toga, 1993; Blasiak et al., 2010; Sergejeva et al., 2015; Rangarajan et al., 2016; but see Slotnick and Brown, 1980), although the precise contexts within which such tests have been conducted differ from our own. Moreover, key differences exist between the brains used to create both reference spaces, including strains and body weights of the source subjects, with *PW* reference space based on the brains of several male Wistar rats ranging 290 ± 16 g in body weight and the *S* atlas series based on a single 315 g, male Sprague-Dawley rat. Kruger et al. (1995) have reported that the midbrain and hindbrain of the Wistar rat is longer in the AP axis than these brain divisions in the Sprague-Dawley rat. On the other hand, Whishaw et al. (1977) have determined empirically that body weight in the laboratory rat is correlated linearly with the location of the Bregma coordinate, a finding supported by a detailed statistical analysis by Slotnick and Brown (1980). Moreover, Paxinos et al. (1985) report that stereotaxic coordinates for their atlas could be applied to animals of differing strains and sexes provided that the animals are of similar surgical body weight to the animals used in their atlas. Nevertheless, it remains unclear whether registration remains accurate at the level of individual transverse sections between the atlases.

Accordingly, we decided to create a semi-automated, quantitative method to examine key landmarks (fiducials) within the tissue sections themselves that form the bases of both atlas reference spaces, as we have noted previously (Khan, 2013; Wells and Khan, 2013). The computer vision algorithm that resulted from this effort utilizes a well-established feature detection technique, known as the Scale-Invariant Feature Transform (SIFT), developed by Lowe (1999, 2004), to detect salient local features in a region of interest (ROI) and the image of the Nissl-stained tissue section documented in each atlas photomicrograph. To match ROIs from one reference atlas space to the other, we implemented a Random Sample Consensus (RANSAC) operation, first developed by Fischler and Bolles (1981), which uses stochastic search to find the affine transformation that yields the largest number of feature correspondences between the images.

We found a striking consensus between the ranked matches returned by the algorithm and the predicted pairings produced by craniometric alignments (summarized in Figure 7). In particular, not only did the algorithm return ranked matches that clustered in the same range as that specified by the craniometric alignment tool, but the differences in the levels between the external (skull-based) and internal (tissue-based) alignments were not more than around 1.6 mm. This distance, if applied as a general “error” value, is probably an overestimate considering that the ROI used to produce this result, the dentate gyrus of the hippocampus, is a relatively large structure that spans many rostrocaudal levels without undergoing drastic variation at the resolution examined here. It is possible that the rostrocaudal difference between the craniometric and computer vision-based alignments would be smaller with selection of ROIs that are more restricted along the rostrocaudal axis, a possibility that will be explored in future expansions of this work.

This deviation notwithstanding, the results demonstrate that there appears to be a strong agreement, under the experimental conditions examined in this study, between craniometric measures of atlas levels for the two reference spaces and the histological features of the underlying tissue sets themselves on which these spaces are based. Future efforts to test this relationship further could include embedding a computational framework within our algorithm that focuses on stable fiducials within the brain tissue sets rather than random features of the test ROI. These fiducials could include white matter tracts and medially located, cytoarchitectonically defined brain structures that are less prone to the variability in appearance that can arise between tissue sections due to differences in their planes of section. Our finding that there is a general consensus between craniometric and histological matchings between the reference spaces is also supported by the statistical analysis conducted by Slotnick and Brown (1980), who found robust correlations (0.68–0.92, mean: 0.81, *n* = 14) of Bregma values with five fiducial points examined within the brains of male albino Holtzmann rats: the most anterior and posterior points of the cerebrum, the genu and splenium of the corpus callosum, and the center of the anterior commissure.

Although our results demonstrate that there is a strong case to be made in using craniometric measures as a first-order means to coarsely align the two reference spaces, a few points concerning our approach are worth noting. First, the search space for our algorithm is limited to an affine transformation between the region of interest and the image as defined by the homography matrix. For tissue that is distorted or warped, further work will be required to generate robust matches, although the results from *Experiment 1a* demonstrate that the algorithm is invariant to some distortion. Second, within brain imaging and analysis, it has been recognized by the community that for many applications and tools developed to streamline registration of images, ground truth is either unavailable or difficult to come by (Prastawa et al., 2005; Bouix et al., 2007; Stevenson et al., 2014). This is due to a variety of reasons, including the difficulty in applying a consistent set of standard operating procedures across the highly variable parts of the brain to permit accurate inter-rater reliability scores that can serve as ground truth. We have experienced such challenges firsthand when attempting to create a basic standard for evaluating neuroanatomical datasets for animal taxa for which poor documentation currently exists (Hughes et al., 2016). A related issue is that the aforementioned 1.6 mm discrepancy that we observed between the craniometric and computer vision-derived values may not be a true “error,” since this assumes that Bregma values serve as ground truth, an assumption that is not always valid but which depends on the types of comparisons being made (e.g., see Richard et al., 2014; Rangarajan et al., 2016). Third, despite the efficacy of our algorithm, there may be an upper limit to its ability to produce accurate matches that is governed by variability between the *PW* and *S* Nissl datasets themselves. As Simmons and Swanson (2009) describe in detail, the major sources of error that investigators encounter when comparing histological data between two brains include intrinsic variability, linear distortion, non-linear distortion, plane of section, and sampling error (see their Figure 1). The processes utilized to generate the Nissl-stained tissue sets for the *PW* and *S* reference spaces were not immune to these types of error, the extent of which will also vary between the two sets of tissue. Finally, the conclusions we draw about the reliability of craniometric alignments must necessarily be constrained by the limited number of atlas levels we evaluated in this study. A near-term goal for expanding this work would be to design and execute a large-scale test to draw statistically valid conclusions about the behavior of the algorithm, hopefully attaining a degree of agreement with an expert that is similar to the agreement between two different experts. Such an effort likely could also benefit from using deep learning approaches (Plis et al., 2014), including deep autoencoders and generative adversarial networks (Goodfellow et al., 2014), as have been applied to human brain MRI image data (e.g., Chen et al., 2016; Moeskops et al., 2017).

### Data migration

In addition to determining the reliability of a basic anteroposterior alignment of the reference spaces, we also evaluated the feasibility of migrating the data representing central microinjection sites between the atlas spaces in the mediolateral and dorsoventral dimensions. Importantly, we sought to ascertain whether subject matter expertise was required for corrections to be made to the basic approach of anisotropically scaling the atlas levels and migrating the data based on stereotaxic coordinates alone. It is striking that all major deviations across the three pairs of *source level*: *destination level* migrations between the reference spaces were in the DV dimension. Although it remains unclear to what extent this trend generalizes for data migrated across all of the registerable levels for *PW* and *S* spaces, its recurrence in our data suggests that this deviation may be a systematic rather than random error when migrating point-source data between the spaces.

While further pairings and analyses are required to support this suggestion, a few possible reasons for this potentially consistent deviation are worth noting here. First, the histological to graphical transformation of the original injection sites, which required the use of a projection microscope, may have produced a distortion in the DV representation of the sites with respect to the size-matched figure of *PW*86 onto which they were projected. If this is the case, then the error may be peculiar to our dataset alone. Second, Swanson (1992) notes that there was a non-linear shrinkage of the tissue for the atlas brain used for *S* space in relation to that used for *PW* space; this shrinkage was greater in the DV dimension and may have been related to compression of the brain along the DV axis during sectioning. Moreover, *PW* space defines the DV zero point as a line tangent to the dorsal surface of the skull, a measure that was not obtained directly for *S* reference space. Some or all of these factors may have contributed to producing slight deviations from the grid of adapted *PW* coordinates designed to fit onto *S* atlas maps.

### Significance of data migration to behavioral studies of the hypothalamus

In the present study, we migrated unpublished central microinjection sites for a published behavioral study (Khan et al., 2004) from *PW* reference space to *S* reference space. For the benefit of the behavioral neuroscience domain experts, a few points about that study are noted here. Stanley et al. (1993a,b) reported that glutamate or its ionotropic receptor agonists, kainic acid, α-amino-3-hydroxy-5-methyl-4-isoxazolepropionate (AMPA), or *N*-methyl-d-aspartate (NMDA); could trigger eating when delivered by microinjection into the rat lateral hypothalamus. Moreover, glutamate receptor antagonists delivered into this region can suppress not only glutamate receptor agonist-elicited feeding, but also feeding triggered by an overnight fast or that triggered by the onset of the nocturnal cycle (Stanley et al., 1996). These results suggested that glutamate is a powerful endogenous controller of food intake. Prompted by reports that protein tyrosine kinases can regulate NMDA receptor function (Wang and Salter, 1994), one of us (AMK) began exploring the possibility that protein tyrosine kinase inhibitors (PTKIs) could alter the food intake triggered by NMDA receptor activation in the LHA. This was later demonstrated in Khan et al. (2004); also see Khan (2002). Specifically, the PTKIs, Tyrphostin A48 (a broad spectrum inhibitor; “*Experiment 4*” in Khan et al., 2004) and PP1 (an inhibitor of Src family tyrosine kinases; “*Experiment 5a*” and “*Experiment 5b*” in Khan et al., 2004), were able to suppress NMDA-elicited eating in a dose-dependent manner (the relevant data from these experiments are reproduced in Figures 13D, 14B).

The data migration we performed in the present study revealed that sites where the PTKIs and NMDA were injected to influence food intake fell within the LHAav, LHAd, LHAma, LHAvm, and an area near the TUl. These regions appear to correspond to the general expanse of the LHA where Src family tyrosine kinases are reportedly expressed (Hirano et al., 1988; Ross et al., 1988; Walaas et al., 1988; Sugrue et al., 1990), and where NMDA receptor subunits are also expressed (Khan et al., 2000). A benefit of this migration is that these behavioral results can be contextualized with other datasets mapped in the same reference space. For example, it remains unclear which cell types within the LHA subregions just described are the substrates that respond to NMDA injections to help mediate the feeding response (or to PTKI inhibitors to suppress this response). Interestingly, several cell types have been mapped in *S* space within these same subregions, including neurons that express the neuropeptides hypocretin/orexin, melanin-concentrating hormone, and neurotensin (Swanson et al., 2005; Watts and Sanchez-Watts, 2007; Hahn, 2010). Thus, migrating the injection sites into a common space where other datasets are mapped allows us to develop new data-constrained hypotheses; in this case, about the possible cell types that might mediate the behavioral effects that we have reported previously. In contrast, many elegant studies that have reported the effects of glutamate microinjections into the LHA remain difficult to contextualize precisely with our current data in the same fashion, since they were not mapped to a reference atlas, yet contain very valuable spatial data that are effectively trapped within the study (e.g., Allen and Cechetto, 1993; Li et al., 2011). An important area for future expansion of brain atlas-based data migration efforts would be devising strategies to migrate and code trapped legacy datasets into extant reference spaces with graphical annotations of their relative positional uncertainty in relation to more precisely mapped datasets, assuming that such efforts are even feasible (also see section 4.6 in Khan, 2013).

### Toward formalizing data migration between stereotaxic reference atlas spaces: basic steps for neuroscientists

#### Data migration as a component of formal scientific transcription

In this study, we demonstrate the stepwise transformation, from tissue section to atlas map, of unpublished hypothalamic injection sites to mapped data points across two distinct and widely used atlas reference spaces for the rat brain (*PW* and *S*). The metamorphosis of a 3-D material object (brain) into a 2-D diagram (map) exemplifies how scientists are frequently engaged in a form of literary “inscription” (which we extend here to also include “transcription”), an idea based on Derrida (1967) and first furnished by Latour and Woolgar (1986) after exploring how thyrotropin-releasing hormone was isolated and assayed from sheep hypothalamic extracts by Guillemin and colleagues (Guillemin and Lemke, 2013). Their sociological study marked the beginnings of treating data transformations in the neurosciences as a formal inscription process, and we emphasize this process here to underscore the importance of establishing a formal laboratory procedure for transforming and migrating spatial datasets between animal brain atlases, complementing efforts now underway, for example, to render human brain imaging datasets interoperable to permit large-scale neurogenomics studies (Medland et al., 2014). As Latour and Woolgar (1986) also argue, the process of inscription is essential in the construction of facts from initial conceptualizations. Accordingly, in the interest of providing a basic operating procedure that can be deployed in most laboratories seeking to migrate various kinds of spatial datasets between *PW* and *S* or vice versa, we offer here a précis of steps generalized from the transformations described in the Methods. For this purpose, we assume that the data to be migrated already exist in published form in one of the two reference spaces. The steps are illustrated here for *PW* to *S* migration, but apply just as readily in the reverse direction. If behavioral neuroscientists keep these steps in mind in relation to their own spatial datasets, they can utilize them to create their own best practice in the lab, compare newer results with those found in previous studies, and/or interrelate data mapped in *PW* reference space with those mapped in *S* space or vice versa.

#### Basic steps for data migration

##### Source:destination alignment

The enterprising behavioral neuroscientist should begin by consulting Figure 2 to identify levels in *S* that correspond to the *PW* levels containing their mapped data. Either there will be *S* levels that match or are closely in register with the relevant *PW* levels by Bregma coordinate, or there will not be. If there are matching levels, the investigator should examine the *S* (destination) levels alongside the *PW* (source) levels, either using physical atlases or the atlas .ai files. The *S* and *PW* levels should appear closely similar based on the structures they contain—due to plane of section differences, etc., it is possible that only a portion of the levels will actually correspond by structure. To proceed with the migration it is important that the regions (for instance, the hypothalamus) of the *PW* levels wherein the mapped data reside should be structurally similar to the same regions in the *S* levels. When assessing structural correspondence, it is wise to give greater consideration to structures that are easily seen in a Nissl stain; whereas structures recognized only by *S* or only by *PW* should be given a lesser weight in the analysis. If the levels are similar in the regions containing data, the neuroscientist may proceed to the steps described below, in *Preparation of the AI Environment*.

If the levels are dissimilar by structure in the data-bearing regions, or if matching levels could not be identified in Figure 2, the neuroscientist is presented with a set of choices. The migration can be abandoned, dissimilar regions can be migrated across despite their differences, or the neuroscientist can explore adjacent *S* levels to identify candidates that do match *PW* by structure in the pertinent regions. For this latter goal, a preliminary analysis of the Figure 2 dot plots, examining how *PW* and *S* levels may correspond based on structural features rather than Bregma coordinates, has been furnished by one of us (Wells, 2017) and may be of use. However, this fiducial analysis awaits validation and further refinement.

##### Preparation of the ai environment

Having identified the *S* levels that match with relevant *PW* levels, the neuroscientist will at this point require access to the .ai files of those levels. These files, at the time of this writing, are available in a few ways. First, *PW* files are downloadable from the publisher's website if the user has purchased a print edition of the atlas, and older CD-ROM-formatted files are available with older *PW* atlas editions. Second, *S* files are now available as open access files for the *S92, S98*, and *S04* editions from https://larrywswanson.com; *S18* files are available in Swanson (2018) as .pdf open access files which can be opened in Adobe Illustrator (AI) software (at the time of this writing, the latest version is AI Creative Cloud).

For any given set of matching levels the following file set-up operations should be performed. In the .ai file for the *PW* level, vector objects associated with the drawing of the *PW* level itself and with its coordinate grid should be grouped and copied into a new .ai file. Similarly, in the .ai file for the *S* level, vector-objects associated with the drawing of the *S* level itself and with its coordinate grid should be grouped and copied into a new layer within the new .ai file. This gives an .ai file containing both the *PW* (source) and *S* (destination) levels, neatly separated into different layers.

However, the levels will at this point be sized differentially. The simplest means of rectifying this is to scale the *S* level, by selecting it and dragging the selection edges, so that its coordinate grid aligns with the *PW* grid—the neuroscientist must take care that the *S* level vectors are grouped before attempting this scaling. The *Zoom* function may be used to increase the accuracy of scaling. It will be necessary to scale *S* independently in the *x*-axis and *y*-axis (i.e., anisotropically). Because *S* uses a derived stereotaxic coordinate system and because the atlas brain used as the basis for *S* was subject to more distorting manipulations (celloidin embedding) than the atlas brains used for *PW*, it is more reasonable to scale *S* to *PW* than vice versa—however, doing so would not be inherently incorrect. The *S* (destination) layer should now be hidden by clicking the *visibility (“eye”) toggle* in the *Layers* window.

Now the mapped data must be imported into the .ai file, using the *Place* command. Assuming it is in a composite image format (i.e., both the mapped data and the underlying map or portion of the map are in the same image), it should be placed into a new layer underneath the *PW* layer, and then aligned and scaled to fit the *PW* level. If there are slight distortions in the image of the map, the image should be aligned so that it matches the *PW* level well in the region where the data occur. The data must then be rendered into vector format, again in a new layer. If the data are point-source, the *Circle Tool* may be used (while holding the **shift** key) to create a circle that is then scaled as appropriate and copied repeatedly, and the copies superimposed over the data. If the data are two-dimensional, then either the neuroscientist can choose to represent them in point-source form, or the *Pencil Tool* may be used to trace them—vectors drawn using the *Pencil Tool* can be edited and readjusted until the fit is correct. While this migration method will be more successful using point-source data, 2-D data migration may be attempted as well with some reduction in accuracy. (Note that we have not yet validated the migration of 2-D data, but only point-source data at this time). The original map image should now be hidden by toggling off its layer's visibility.

Having followed these steps, the neuroscientist possesses a series of .ai files, one for each set of matching *PW* and *S* levels. Each file contains four layers. The *S* and *PW* levels are aligned by their stereotaxic grids, the original mapped data are aligned with the *PW* level, and a vector representation of the data appears over the original. Migration can begin.

##### Migration

At this point, migrating the data based solely on stereotaxic coordinates is as simple as toggling off the visibility on the *PW* (source) level and the original map image, and toggling on the visibility on the *S* (destination) level, in each .ai file in the series. However, it will probably be necessary to take additional steps to account for finer differences in the exact disposition of structures between the *PW* and *S* levels.

##### Refinement

The neuroscientist should toggle on visibility to the *PW* level and study the relationship between the vector-formatted mapped data and nearby structures on *PW*, paying particular attention to any structures that are easily identified in Nissl-stained material, and paying less attention to any structures that are recognized only by *PW*. Then the visibility should be toggled off for *PW* and on for *S*, and the relationship between the data and nearby structures on *S* examined, noting any differences. This process should be repeated until the neuroscientist has a reasonable idea of the differences between the data's relationship to *PW* structures and their relationship to *S* structures. The original map image can be referenced for enhanced accuracy in this process.

The neuroscientist should then nudge the vector-formatted data until their positions vis-à-vis *S* structures matches their original positions vis-à-vis *PW* structures—the map image will be crucial here for assessing the original positions on *PW*. If the data are point-source, each vector circle should be nudged singly (unless some of the circles are very closely clustered; such clusters may be nudged as a group). Figures 9–11 may be referenced for an example of this process, which will be highly individualized for each set of mapped data migrated. If the data are two-dimensional and not represented as point-source simplifications, it may well be necessary to scale them in addition to nudging. Here problems may arise: increasing the accuracy of the positioning of the migrated data may require significantly distorting their original shape, and increases in positional accuracy in one region of *S* may come only at the price of decreases in positional accuracy in another region. Resolving these difficulties would require the exercise of professional judgment regarding where accuracy may be sacrificed on a case-by-case basis. It is recommended that any and all transformations made to the data during migration be documented thoroughly.

## Future directions

Having described the basic steps of data migration, it is useful to consider the benefits of such a procedure in relation to the references spaces themselves. One benefit of registering reference spaces is that other brain atlases may already be registered to one or more of these spaces. For example, Leergaard et al. (2003) registered their manganese-enhanced MRI datasets with a digital 3-D reconstruction of Swanson reference space. A number of investigators have registered rat MRI or fMRI data with a Paxinos and Watson reference space (Schweinhardt et al., 2003; Schwarz et al., 2006; Lu et al., 2010; Johnson et al., 2012; Wisner et al., 2016). A new reference space for the mouse brain has been created that allows for interoperability among various online mouse brain resources within a common framework. Called *Waxholm Space* (“*WHS*”; Johnson et al., 2010; Bowden et al., 2011; Hawrylycz et al., 2011), *WHS* has also been created recently for the adult male Sprague-Dawley rat (Papp et al., 2014). The registration of multiple atlases with one another allows for greater interoperability between datasets (Toga and Thompson, 2001). This form of *model-to-model registration* (Zitová and Flusser, 2003) serves to ensure the lasting preservation and more widespread use of the hard-earned datasets produced from time- and labor-intensive experiments. Developing more robust pipelines to enable migration of mapped data across multiple reference systems will be a valuable means to further integrate the work of numerous investigators. One challenge that still remains is to enable data migration across reference spaces in a manner that takes into account differences in the boundary conditions of brain sub-regions in each reference space and across different scales (e.g., see Martone et al., 2008; Bohland et al., 2009).

A related challenge is the task of registering datasets in *PW* or *S* reference spaces, which are based on a “flat skull” orientation (i.e., no DV difference in the positions of a probe touching the Bregma or Lambda suture intersections), with older reference spaces such as the widely used de Groot rat atlases of the rat forebrain (de Groot, 1959a) and hypothalamus (de Groot, 1959b), and those of the rat brain by Pellegrino and colleagues (Pellegrino and Cushman, 1967; Pellegrino et al., 1979). These atlases utilized brains that were tilted in the stereotaxic frame to create a horizontal plane through the anterior and posterior commissures (+5.0 mm above the interaural line). The transverse plane atlas maps produced by this latter orientation differ markedly in the regional and sub-regional cytoarchitectonic boundaries they contain from those produced using a flat skull reference plane. A future expansion of the efforts set forth here could be to utilize computational methods to bring these atlases in register with *PW* and *S*, so data from valuable studies utilizing these reference spaces (e.g., see Chiappa et al., 1977) can be contextualized with *PW* and *S* datasets.

## Concluding remarks

In this study, we have demonstrated the first-order alignment and migration of point-source data consisting of central microinjection sites in the hypothalamus to the *S* atlas space from the *PW* space in three dimensions. It is anticipated that the approach for data migration outlined in this study will be useful to neuroscientists seeking to contextualize their datasets in these reference spaces with one another to generate new insights about structure-function relations in the brain. It should also prove useful as a starting point toward further work in atlas-based registration of experimental tissue.

## Author contributions

AK and OF conceived of this project, with AK supervising its neuroanatomy component and OF supervising the computer vision component. AK constructed the alignment tool and Cleveland plots, and performed the data migration analysis. JP developed the computer vision algorithm and performed the experiments to test its efficacy, with guidance from OF and AK. CW conducted the transformation and migration of the central microinjection data, with guidance from AK. OF wrote the pseudocode in Figure 1, with feedback from JP. AK wrote the manuscript, with contributions from all of the authors.

### Conflict of interest statement

The authors declare that the research was conducted in the absence of any commercial or financial relationships that could be construed as a potential conflict of interest.

## List of abbreviations

### Paxinos and Watson nomenclature

3V, third ventricle; A11, dopamine cells; ACo, anterior cortical amygdaloid nucleus; AHA, anterior hypothalamic area, anterior part; Arc, arcuate hypothalamic nucleus; BAOT, bed nucleus of the accessory olfactory tract; DA, dorsal hypothalamic area; DM, dorsomedial hypothalamic nucleus; DMD, dorsomedial hypothalamic nucleus, dorsal part; F, nucleus of the fields of Forel; ic, internal capsule; LGP, lateral globus pallidus; LH, lateral hypothalamic area; MCLH, magnocellular nucleus of the lateral hypothalamus; MeAD, medial amygdaloid nucleus, anterior dorsal part; MEE, medial eminence, external layer; MEI, medial eminence, internal layer; MePD, medial amygdaloid nucleus, posterodorsal part; MePV, medial amygdaloid nucleus, posteroventral part; mfb, medial forebrain bundle; MGP, medial globus pallidus; mt, mammillothalamic tract; MTu, medial tuberal nucleus; opt, optic tract; Pa, paraventricular hypothalamic nucleus; Pe, periventricular hypothalamic nucleus; PeF, perifornical nucleus; PH, posterior hypothalamic nucleus; RCH, retrochiasmatic area; Re, reuniens thalamic nucleus; Rt, reticular thalamic nucleus; SI, substantia innominata; SO, supraoptic nucleus; SOR, supraoptic nucleus, retrochiasmatic part; sox, supraoptic decussation; SubI, subincertal nucleus; SubV, submedius thalamic nucleus, ventral part; TC, tuber cinereum area; Te, terete hypothalamic nucleus; VM, ventromedial thalamic nucleus; VMH, ventromedial hypothalamic nucleus; VRe, ventral reuniens thalamic nucleus; ZI, zona incerta; ZIV, zona incerta, ventral part.

### Swanson nomenclature

The nomenclature system here, based on Swanson (2015), consists of *standard term names*, followed by the last name of the author and the year in which the *standard term name* was first used *(author, date)*. Thus, a ***standard term*** is defined as *standard term name* + *(author, date)*. Terms named after 1840, but where the first use of the term has not been traced, are indicated as “*standard term name (>1840)*” to note this fact. A full listing of the sources of the *(author, date)* portions of each ***standard term*** can be found in the References, but note that these specific sources were not necessarily consulted directly by us, but are cited in Swanson (2015, 2018).

AHN, ***anterior hypothalamic nucleus (***>***1840)***; ARH, ***arcuate hypothalamic nucleus (***>***1840)***; BA, ***bed nucleus of accessory olfactory tract (Scalia and Winans, 1975)***; COAa, ***cortical amygdalar area anterior part (***>***1840)***; COApm, ***cortical amygdalar area posterior part medial zone (***>***1840)***; cpd, ***cerebral peduncle (Tarin, 1753)***; ***DMH, dorsomedial hypothalamic nucleus (***>***1840)***; em, ***external medullary lamina (***>***1840)***; I, ***internuclear hypothalamic area (Swanson, 2004)***; int, ***internal capsule (Burdach, 1822)***; LHA, ***lateral hypothalamic area (Nissl, 1913)***; LHAad, ***lateral hypothalamic area anterior group anterior region dorsal zone (Swanson, 2004)***; LHAav, ***lateral hypothalamic area anterior group anterior region ventral zone (Swanson, 2004)***; LHAjp, ***lateral hypothalamic area middle group medial tier juxtaparaventricular region (Swanson, 2004)***; LHAsfa, ***lateral hypothalamic area middle group perifornical tier subfornical region anterior zone (Swanson, 2004)***; ME, ***median eminence (Tilney, 1936)***; ***MEAad, medial amygdalar nucleus anterodorsal part (***>***1840)***; MEApd, ***medial amygdalar nucleus posterodorsal part (***>***1840)***; MEApv, ***medial amygdalar nucleus posteroventral part (***>***1840)***; mtt, ***mammillothalamic tract (Kölliker, 1896)***; opth, ***hypothalamic optic tract (Swanson, 2015)***; PH, ***posterior hypothalamic nucleus (***>***1840)***; pofh, ***hypothalamic postcommissural fornix (Swanson, 2015)***; PR, ***perireuniens nucleus (Brittain, 1988)***; PVH, ***paraventricular hypothalamic nucleus (***>***1840)***; PVi, ***periventricular hypothalamic nucleus anterior part intermediate zone (Swanson, 2018)***; RE, ***nucleus reuniens (Malone, 1910)***; SBPV, ***subparaventricular zone (Watts et al., 1987)***; SI, ***innominate substance (Schwalbe, 1881)***; SOp, ***supraoptic nucleus principal part (Swanson, 2018)***; ste, ***endbrain terminal stria (Swanson, 2015)***; suph, ***hypothalamic supraoptic decussations (Swanson, 2018)***; TUi, ***lateral hypothalamic area middle group lateral tier tuberal nucleus intermediate part (Swanson, 2004)***; TUsv, ***lateral hypothalamic area middle group lateral tier tuberal nucleus subventromedial part (Swanson, 2018)***; V3h, ***hypothalamic part of third ventricle principal part (Swanson, 2015)***; vlt, ***ventrolateral hypothalamic tract (Swanson, 2004)***; VM, ***ventral medial thalamic nucleus (***>***1840)***; VMH, ***ventromedial hypothalamic nucleus (***>***1840)***; ZI, ***zona incerta (***>***1840)***.
